# Circular RNA cESRP1 sensitises small cell lung cancer cells to chemotherapy by sponging miR-93-5p to inhibit TGF-β signalling

**DOI:** 10.1038/s41418-019-0455-x

**Published:** 2019-11-14

**Authors:** Weimei Huang, Yunchu Yang, Jingfang Wu, Yuchun Niu, Yao Yao, Jian Zhang, Xiaoxian Huang, Shumei Liang, Rui Chen, Size Chen, Linlang Guo

**Affiliations:** 1 Department of Pathology, Zhujiang Hospital, Southern Medical University, Guangzhou, China; 2 Department of Oncology, The First Affiliated Hospital of Hebei North University, Zhangjiakou, China; 3 Department of Pathology, Peking University Third Hospital, Beijing, China; 4 Department of Oncology, Zhujiang Hospital, Southern Medical University, Guangzhou, China; 5 Clinical Laboratory, Gushang Hospital of Guangxi Zhuang Autonomous Region, Nanning, China; 6 Department of Oncology, The First Affiliated Hospital of Guangdong Pharmaceutical University, Guangzhou, China

**Keywords:** Small-cell lung cancer, Gene expression

## Abstract

Circular RNAs (circRNAs) are novel RNA molecules that play important roles in chemoresistance in different cancers, including breast and gastric cancers. However, whether circRNAs are involved in the response to chemotherapy in small cell lung cancer (SCLC) remains largely unknown. In this study, we observed that cESRP1 (circular RNA epithelial splicing regulatory protein-1) expression was significantly downregulated in the chemoresistant cells compared with the parental chemosensitive cells. cESRP1 enhanced drug sensitivity by repressing miR-93-5p in SCLC. Cytoplasmic cESRP1 could directly bind to miR-93-5p and inhibit the posttranscriptional repression mediated by miR-93-5p, thereby upregulating the expression of the miR-93-5p downstream targets Smad7/p21(CDKN1A) and forming a negative feedback loop to regulate transforming growth factor-β (TGF-β) mediated epithelial-mesenchymal transition. Furthermore, cESRP1 overexpression and TGF-β pathway inhibition both altered tumour responsiveness to chemotherapy in an acquired chemoresistant patient-derived xenograft model. Importantly, cESRP1 expression was downregulated in SCLC patient tissues and was associated with survival. Our findings reveal, for the first time, that cESRP1 plays crucial a role in SCLC chemosensitivity by sponging miR-93-5p to inhibit the TGF-β pathway, suggesting that cESRP1 may serve as a valuable prognostic biomarker and a potential therapeutic target in SCLC patients.

## Introduction

Lung cancer remains one of the most common causes of cancer-related mortality worldwide, with small cell lung cancer (SCLC) accounting for ~15% of all lung cancer cases [1, 2]. The high death rate of SCLC patients is directly related to the fact that most patients eventually develop resistance to platinum-based chemotherapy and ultimately die from their disease [3]. Thus, there is a drastic need to identify new therapeutic targets or treatments that restore chemosensitivity and improve tumour control in relapsed chemoresistant SCLC patients.

Circular RNAs (circRNAs) are naturally occurring members of the noncoding transcriptome and have a covalently closed loop structure [4]. The functional roles of circRNAs have been well characterised to date, such as in mesenchymal stem cell identity maintenance [5], differentiation [6], development [7], and oncogenesis [8, 9]. CircRNAs can influence various cellular processes including proliferation, cell cycle progression, and cell apoptosis [10, 11]. More meaningfully, they can act as oncogenes or tumour suppressors through diverse mechanisms to regulate tumour progression [9, 12] and tumour resistance to chemotherapy [13, 14]. However, the circRNAs involved in SCLC chemoresistance remain largely unknown.

Transforming growth factor-β (TGF-β) is one of the most prominent molecules involved in cancer progression, including SCLC [15, 16]. During cell development and carcinogenesis, TGF-β ligand activation results in the phosphorylation of TGF-β receptor type I (TβRI), Smad2, and Smad3 (Smad2/3) [17]. Subsequently, phosphorylated Smad2/3 form complexes with Smad4 and translocate into the nucleus, binding to specific DNA sequence motifs and regulating the transcription of target genes, including Smad7 [18, 19]. Smad7 can act as negative feedback regulator of the TGF-β-mediated signalling by inhibiting TβRI-regulated Smad2/3 phosphorylation or impeding the binding of Smads complexes to DNA sequences [20, 21]. In addition to regulating Smad7, the p-Smad2/3-Smad4 complex can also interact with additional transcriptional regulators in the nucleus to transactivate downstream target genes of TGF-β, such as p21(CDKN1A) [22–24]. Upon TGF-β signalling activation, a series of processes occur that can lead to cancer development, and TGF-β signalling can increase drug resistance in tumour cells by mediating epithelial-to-mesenchymal transition (EMT) [25, 26]. Blocking the TGF-β signalling pathway can reverse chemoresistance in tumour cells [27]. In several cancers, such as prostate cancer, glioblastoma, and lung cancer, the TGF-β receptor I inhibitor galunisertib can sensitise cancer cells to anticancer drugs by suppressing TGF-β-mediated EMT [28–30]. It has been reported that p21(CDKN1A) is upregulated by TGF-β-mediated pathways and can repress TGF-β-induced features of EMT by interfering with TGFBR2 expression or reversing twist-mediated E-cadherin promoter repression [31, 32]. However, whether p21(CDKN1A) plays a role in chemoresistance through TGF-β-mediated EMT remains unknown. Recently, noncoding RNAs, including miRNA and circRNA, have been shown to promote chemoresistance via the TGF-β-induced EMT pathway [33–36]. However, to date, no studies have reported whether circRNAs can affect SCLC chemoresistance by repressing TGF-β-induced EMT.

In this study, we screened for circRNAs exhibiting differentially expression between the parental SCLC cell line H69/H69AR and identified a novel circRNA derived from the ESRP1 (RBM35A) gene locus (cESRP1). Low expression of cESRP1 in patients with SCLC was positively associated with poor survival. Further investigation showed a critical role for cESRP1 in inhibiting TGF-β/Smad signalling-induced EMT. We revealed, for the first time, a novel interaction between cESRP1 and a protein complex known to be involved in the negative feedback regulation of TGF-β/Smad signalling activation, establishing cESRP1 as an emerging circRNA that is functionally important in SCLC chemosensitivity.

## Materials and methods

### Tumour specimens and primary cell cultures

Patient-derived paraffin-embedded tumour sections were collected from the First Affiliated Hospital of Hebei North University and the Minzu Hospital of Guangxi Zhuang Autonomous Region for use in this study, which was approved by the appropriate ethics committee. Informed consent was obtained from all patients before specimen collection. RNA was extracted from the paraffin-embedded tissue samples to assess the RNA levels of target molecules using an FFPE RNA kit (GBCBIO, Guangzhou, China), and the primers used are provided in the Supplemental Information. The three cell lines (NCI-H69, NCI-H69AR, and NCI-H446) used in this study were purchased from the American Type Culture Collection (ATCC, USA) and cultured in RPMI medium (HyClone; Thermo Scientific, CA, USA) supplemented with 10% fetal bovine serum (FBS; Gibco, New York, USA) in a humidified atmosphere containing 5% CO_2_ at 37 °C. Cell line identity was commercially authenticated via short tandem repeat profiling, which was performed by the Cellcook Biology Company of China. The drug-resistant subline H446DDP was established in our laboratory by culturing H446 cells in gradually increasing concentrations of cisplatin (up to 0.5 μg/ml) for 12 months and was maintained in complete medium containing cisplatin. The primary cell line PDC1-S was produced from fresh xenograft tumour tissue from patient-derived xenograft (PDX)1-naive mice, and the cell line PDC1-R was produced from PDX1-chemoresistant mice; Both cell lines were maintained in Dulbecco’s modified Eagle’s medium (DMEM)/F-12 (Gibco, New York, USA) supplemented with 10% FBS (Gibco, New York, USA). The extraction steps used to process primary cells and the reagents used are specifically listed in the *Solid tissue disaggregation* section. The cell lines used in this study were not contaminated with mycoplasma.

### circRNA expression profiles

Experimental technology was provided by the Shanghai Kangcheng Biological Company (China). Briefly, H69 and H69AR cells were used for circRNA microarray assays. Total RNA was extracted from cell lysates and evaluated for quality by agarose gel electrophoresis. Two micrograms of total RNA were treated with RNase R. After sample labelling, hybridisation, and washing, the samples were analysed using circRNA chips (Arraystar Human circRNAs chip; Arraystar, Rockville, MD, USA). Exogenous RNAs developed by the External RNA Controls Consortium (Applied Biosystems, USA) were used as controls.

### Cell counting kit-8 assay and the determination of 50% inhibitory concentration (IC50) values

Cells in complete growth medium were inoculated into a 96-well tissue culture plate at a density of 3000–12,000 cells per well. After 24 h of culturing, growth medium containing chemotherapeutic drugs, including cisplatin (cisplatin injection; Shandong, China), etoposide (Vepesid; Bristol-Meyers Squibb, Australia), and doxorubicin (Hisun Pfizer; Hangzhou, China) was added to the wells. Wells containing drug-free growth medium were used as controls. Then, the plate was incubated for 24 h before assessing cell viability. Luminescence analysis was performed according to the instructions of the CCK8 manufacturer (Dojindo, Japan), and the 50% inhibitory concentration (IC50) values of the drugs were calculated using Graphpad.

### RNA isolation, treatment with RNase R, and quantitative real-time PCR (qRT-PCR)

Total RNA was extracted from cells and tumour samples using RNAiso Plus* (Takara, Japan) according to the manufacturer’s instructions. Cytoplasmic and nuclear RNA was isolated using a Nuclear/Cytoplasmic Isolation Kit (BioVision, San Francisco, USA) according to the manufacturer’s instructions. For RNase R treatment, 1500 ng of total RNA was incubated for 30 min at 37 °C with or without 2 U/μg RNase R (Epicentre Technologies, Madison, WI, USA). cDNA was synthesised using a Fast Quant RT Kit (TIANGEN BIOTECH, Beijing, China) according to the manufacturer’s instructions. Then, quantitative real-time PCR (qRT-PCR) was performed using 2 × Talent qPCR PreMix (TIANGEN BIOTECH, Beijing, China) according to the manufacturer’s guidelines with a Bio-Rad CFX Connect instrument (Bio-Rad, USA). The relative RNA expression levels were analysed using the 2^−ΔΔCt^ method, with β-actin used as an internal reference. The primers and RNA sequences used for qRT-PCR are shown in the Supplemental Information.

### Fluorescence in situ hybridisation (FISH)

FITC-labelled miR-93-5p and Cy3-labelled cESRP1 probes were designed and commercially synthesised by GenePharma (Shanghai, China). The probe sequences are provided in the Supplemental Information. A fluorescence in situ hybridisation (FISH) kit (RiboBio, Guangzhou, China) was used to detect probe signals according to the manufacturer’s instructions after culturing cells for 24 h. To determine the cESRP1 status of PDX tumours, 4-μm-thick sections were cut from paraffin-embedded blocks and then processed, hybridised, and analysed. Cell nuclei were stained with 4’,6-diamidino-2-phenylindole (DAPI; Life Technologies, Carlsbad, CA, USA), and images were acquired using an LMS 880 confocal microscope (Carl Zeiss, Germany).

### Western blotting (WB)

Cell lysates were prepared using RIPA buffer (CWBIO, Beijing, China) supplemented with a phosphatase inhibitor cocktail (CWBIO, Beijing, China) and a protease inhibitor cocktail (CWBIO, Beijing, China). Protein concentrations were determined using a Bicinchoninic acid (BCA) Protein Assay Kit (Beyotime Biotechnology, Shanghai, China). Lysates were boiled in SDS-PAGE loading buffer (Beyotime Biotechnology, Shanghai, China) for 10 min at 95 °C, after which 30–50 μg of each protein sample was separated by SDS-PAGE and then electrotransferred to PVDF membranes (Millipore, IPVH00010; Billerica, MA, USA). The membranes were then blocked with 5% bovine serum albumin (BSA; MRC BIOTECH, Beijing, China). Finally, the blots were incubated with the appropriate primary antibody (Supplemental Information) overnight at 4 °C and followed by the corresponding HRP-conjugated secondary IgG antibody for 1 h at room temperature. Chemiluminescence WB reagents (Millipore Corporation, Billerica, MA, USA) were used to detect immunocomplexes. β-actin served as the loading control in this study.

### Pulldown assay using a biotinylated miRNA

The capture of miR-93-5p-bound competing endogenous RNAs (ceRNAs) in a pulldown assay using biotinylated miR-93-5p was performed as previously described [37]. Briefly, 5–10 × 10^6^ cells were transfected with 40 µg of a biotinylated miR-93-5p mimic (RiboBio, Guangzhou, China) using Lipofectamine™ 3000 Transfection Reagent (Invitrogen, CA, USA) according to the manufacturer’s instructions. A biotinylated negative mimic served as a control. The cells were harvested for the pulldown experiment 24 h after transfection. Biotin-coupled RNA complexes were pulled down by incubating the cell lysates with streptavidin-coated magnetic beads (Dynabeads®M-280, Life Technologies, USA & Canada) on a rotator at 4 °C overnight. The next day, the M-280 Dynabead-miRNA mixture was washed with a lysis buffer five times, after which the bound RNA was treated with TRIzol for RNA extraction and purified using a phenol:chloroform:isoamyl alcohol mixture (Millipore Corporation, Billerica, MA, USA). Finally, purified RNA was analysed by qRT-PCR.

### Histological evaluation and immunohistochemical staining

Tissue samples embedded in paraffin were cut into serial 4-µm-thick sections, and then the sections were baked at 65 °C for 2 h, deparaffinized in three changes of xylene and rehydrated through 5-min incubations in 100, 95, 85, and 75% ethanol solutions. Then, the sections were rinsed in phosphate-buffered saline (PBS) for 5 min. For histological examination, sections were stained with haematoxylin and eosin (LEAGENE, Beijing, China) for 3–10 min. Immunohistochemical staining was performed using the Streptavidin-Peroxidase Kit (ZSBIO, Beijing, China) according to the manufacturer’s instructions. Briefly, antigen retrieval was performed using Sodium Citrate + EDTA buffer at 95–100 °C for 8 min, after which the sections were cooled to room temperature. After rinsing in PBS three times for 5 min each time, endogenous peroxidase activity was blocked using 1% H_2_O_2_ for 15 min, followed by blocking of the nonspecific binding sites with goat serum for 30 min at room temperature. Then, the sections were incubated with a specific primary antibody overnight at 4 °C. The next day, the sections were rinsed in PBS three times for 5 min each time, which was followed by an incubation with a biotinylated secondary antibody for 30 min at room temperature. Each section was rinsed in PBS three times and incubated with streptavidin-conjugated HRP for 30 min at room temperature. HRP activity was detected using diaminobenzidine tetrahydrochloride (DAB), and nuclei were distinguished by haematoxylin staining. Dehydration was accomplished by incubating the sections in 75, 85, 95, and 100% ethanol solutions for 5 min for each solution. In the last step, the sections were cleared in xylene and sealed with neutral gum. Images were acquired using a Leica DM2500 microscope (Leica, Germany). A detailed list of the antibodies used is included in the Supplemental Information.

### Immunofluorescence assays

Cells were seeded on slides for 24 h and then treated as indicated in the figures. The cells were fixed in 4% paraformaldehyde for 30 min and rinsed in PBS three times for 5 min each time. Subsequently, the slides were incubated in 0.5% Triton X-100 for 15 min at room temperature for permeabilization, and nonspecific protein binding was blocked by incubating the cells with goat serum for 30 min at room temperature. Then, the cells were incubated with a primary antibody overnight at 4 °C. After rinsing in PBS three times for 5 min each time, the cells were incubated with a dye-conjugated secondary antibody for 1 h at room temperature. Then, the cells were washed three times in PBS and incubated with DAPI for 10 min at 37 °C before being washed in PBS three times for 5 min each time. An anti-fluorescence quencher (Solarbio, Beijing, China) was used in the last step, and images were acquired using a confocal microscope. A detailed list of the antibodies used is provided in the Supplemental Information.

### RNA immunoprecipitation (RIP) assay

RIP assays were performed using a Magna RIP™ RNA-Binding Protein Immunoprecipitation Kit (Millipore Corporation, Billerica, MA, USA) as previously described [38]. Briefly, SCLC cells were cultured in 75-cm^2^ cell culture flasks for 24 h and then harvested and lysed using RIP lysis buffer. Five micrograms of argonaute 2 (Ago2) antibody or normal rabbit lgG (Negative control; Millipore, MA, USA) was pre-incubated with Magnetic beads to form a magnetic bead–antibody complex; then, cell lysates were incubated with the magnetic bead–antibody complex overnight at 4 °C. Subsequently, the RNA in the immunoprecipitates was purified according to the kit protocol from the manufacturer. The extracted RNA was then analysed by qRT-PCR.

### Transient transfection and luciferase reporter assays

An miRNA mimic and an miRNA inhibitor were purchased from RiboBio. siRNAs specific for cESRP1 but not for mESRP1 as well as a full-length cESRP1 clone in the vector pcDNA3.1 were purchased from GenePharma. The cESRP1 overexpression or cESRP1-siRNA constructs were packaged into adenovirues by GenePharma. Plasmids and oligonucleotides were transfected using Lipofectamine^TM^ 3000 Transfection Reagent (Invitrogen, CA, USA). For the luciferase reporter assays, the pREL-RB-TGF-beta plasmid was commercially synthesised by RiboBio and was used to verify TGF-β transcriptional activity. GV272 luciferase reporter constructs containing a wild-type (WT) or mutant cESRP1 sequences were obtained from the Shanghai GeneChem Corporation of China and used to evaluate the ceRNA activity of cESRP1, where the GV045 plasmid used as an internal control. SCLC cells were transiently transfected with the indicated constructs and a Renilla luciferase plasmid as an internal control. For the TGF-β activity assay, 24 h after transfection, cells were serum starved for 8 h before stimulation with 10 ng/ml of human recombinant TGF-β1 (Selleckchem, Houston, TX, USA), with luciferase activities were quantified 14 h later using a dual-luciferase assay (Promega, Germany). For the ceRNA activity assay, 24 h after transfecting cESRP1 clone plasmids, miR-93-5p mimics were reintroduced and the cells were cultured for an additional 12 h, followed luciferase activity measurement. The luciferase values shown in the figures are representative of independent transfection experiments that were performed independently at least three times. siRNA sequences are provided in the Supplemental Information.

### Establishment of cell lines with stable cESRP1 knockdown or overexpression

Construction of the pLVX-puro plasmids and packaging of the cESRP1 overexpression and cESRP1 knockdown lentiviruses were performed at GenePharma. SCLC cells were seeded in 24-well plates. When the cell fusion rate reached 40–60%, an MOI (multiplicity of infection) of 50 of lentivirus was added and cells were cultured in complete medium containing 1 µg/ml polybrene for 12–24 h. The transfected cells were maintained in complete medium containing 2 µg/ml puromycin (Solarbio, Beijing, China).

### miRNA expression profiles

The microarray assay was performed by LC Sciences (Texas, USA). Briefly, 2–5 μg of total RNA was extracted and an oligonucleotide tag was then ligated to the poly(A) tails for later fluorescent dye staining. Subsequently, hybridisation was performed on a μParafloTM microfluidic chip using a micro-circulation pump (Atactic Technologies) [39] according to the instructions of manufacturer. PGR (photogenerated reagent) chemistry was used to detect the probes. After hybridisation, the detection was performed using fluorescence labelling with tag-specific Cy3 and Cy5 dyes, and hybridisation images were collected using a laser scanner (GenePix 4000B, Molecular Device). The ratio of the two signals (log_2_ transformed, balanced) and *p* values of the *t*-test were calculated, with significantly different signals identified as those with *p* values of less than 0.01.

### Flow cytometry analysis

To analyse cell apoptosis, after drug treatment, cells were harvested by digestion with trypsin (Gibco, New York, USA) and washed three times with PBS containing 2% FBS and 2% BSA. Then, the cells were incubated with Annexin-V-APC (eBioscience, Thermo Fisher Scientific, CA, USA) for 30 min at 4 °C. The cells were rinsed three times in PBS, followed by a 5-min incubation in Fixable Viability Dye eFluor™ 780 (eBioscience, Thermo Fisher Scientific, CA, USA). For cell cycle analysis, the cells were fixed in 70% ice-cold ethanol for 2 h and stained with propidium iodide (PI, Keygen, Jiangsu, China) in the presence of RNase A (Qiagen, Germany). Fluorescence intensity was measured using a FAC Scan (BD Biosciences, USA). Apoptotic cells were defined as cells with Annexin V-positive staining. The percentages of cells in the G0-G1, S, and G2-M cell cycle phases were counted and compared.

### Animal experiments

#### SCLC cell line-derived tumour xenograft model

Research involving animals was performed in compliance with the policies of the animal ethics committee of the Southern Medical University of China. Female BALB/c nude mice aged 3–4 weeks were used for tumour implantation experiments. SCLC cells were resuspended in 100 μl of PBS and injected subcutaneously into the flanks of the nude mice. At 7–10 days after implantation, when the tumours became palpable with a diameter of ~5 mm, drugs were intraperitoneally (i.p.) or intravenously (i.v.) injected. For the in vivo administration of the chemotherapeutic drugs cisplatin and etoposide, cohorts of tumour-bearing animals were treated weekly with cycles of cisplatin (cisplatin injection; Shandong, China; 2.5 mg/kg, i.p. injection on day 1) and etoposide (Vepesid; Bristol-Meyers Squibb, Australia; 4 mg/kg, i.p. injection on days 1–3). For the miRNA antagomir treatment, 50 μl of the miR-93–5p or control antagomir (diluted in PBS to 2 mg/ml, administered on days 1–3, weekly) was i.v. administered, and LY2157299 (2 mg/kg, days 1–3, weekly) was i.p. injected alone or in combination with the chemotherapeutic drugs (cisplatin, 1.5 mg/kg, day 1, i.p.; and etoposide, 3 mg/kg, days 1–3, i.p.). Tumour size and mouse body weight were measured with electronic calipers and an electronic scale, respectively, every 3-4 days. Tumour volume was calculated using on the following formula: (length × width^2^)/2.

#### Establishment of the PDX model

PDX models using samples derived from SCLC patients have been described elsewhere [40–42]. To collect fresh tissues, patients treated for SCLC at the Guangdong Provincial People’s Hospital and the Zhujiang Hospital of Southern Medical University provided informed consent to the Institutional Review Board. All mouse studies were conducted according to Institutional Animal Care and Use Committee (IACUC)-approved animal protocols in accordance with the Southern Medical University’s institutional guidelines. The diagnosis of SCLC was confirmed by a pathologist. To establish a PDX model (P0), SCLC tumour samples (primary surgical tumour specimens or metastatic lymph node resection specimens) were cut into 3–4 mm pieces and subcutaneously transplanted within 4 h after surgical removal into 3–5 severely immunodeficient B-NDG® mice (BIOCYTOGEN, Beijing, China). The mice were observed daily to assess tumour growth and animal health status. Palpable tumours were measured weekly with electronic callipers until the tumour size exceeded 1000–1500 mm^3^. At this time, the animals were euthanized, and the tumours were removed. Subsequently, scalpel-dissected xenograft fragments were either immediately implanted into new NDG® mice for passaging, digested into single-cell suspensions and cryopreserved in liquid nitrogen for later passaging, fixed in 4% paraformaldehyde (YongJin Biotech, Guangzhou, China) for pathological analysis, or flash-frozen in liquid nitrogen for molecular analysis.

#### Solid tissue disaggregation

Solid tissue samples collected from mouse xenografts were mechanically and enzymatically disaggregated into single-cell suspensions as previously described [43, 44]. Briefly, the solid tissue samples were minced with scissors into small (1 mm^3^) fragments and incubated for 1.5 h at 37 °C in a phosphate buffer (pH 7.0) with 1 mg/ml of collagenase type I (Solarbio, Beijing, China), 1 mg/ml of collagenase type II (Solarbio, Beijing, China), and 50 units/ml DNase I (Sigma, MO, USA) with occasional vibration to achieve enzymatic disaggregation. Then, an equal volume of the phosphate buffer was added, after which the cells were resuspended by pipetting and then filtered through a 100-µm nylon mesh. Single cells were used for culture and cryopreservation.

#### In vivo generation of acquired resistance to cisplatin/etoposide (C/E)

To establish a chemoresistant subcutaneous PDX model, we performed a previously described procedure [42] after the tumours grew to a size of ~50 mm^3^. Cohorts of animals were treated once every ten days with cycles of cisplatin (2.5 mg/kg, i.p. injection on day 1) and etoposide (4 mg/kg, i.p. injection on days 1–3), which were intraperitoneally injected at least six times during each round of treatment. When the tumour volume reached ~500 mm^3^, the mice were euthanized, and the tumours were removed. The drug-treated tumours were divided into small fragments and engrafted into the next generation of mice following the same method of cisplatin/etoposide (C/E) or vehicle treatment until chemoresistant tumours developed.

#### Chemosensitivity testing of the PDX model

The chemotherapeutic response of the PDX model tumours was determined by digesting tumours into single-cell suspensions and subcutaneously injecting an equal number of cells into a large number of B-NDG® mice. When the tumours reached a palpable size (5 mm), the mice were randomised into treatment and vehicle control groups. To examine the tumour response to first-line chemotherapeutic drugs for SCLC therapy, the following drugs and treatment modalities were used: cisplatin at a dosage of 2.5 mg/kg/d, qd 1, i.p. injection; and etoposide at a dosage of 4 mg/kg/d, qd 1–3, i.p. injection. Doses and schedules were chosen according to previous experience with animal studies and represent the maximum tolerated or efficient doses in our investigation.

To assess the sensitivity of tumours to chemotherapy after a combination treatment with chemotherapeutic drugs and the TGF-β inhibitor LY2157299 (Selleck, Houston, TX, USA), the following treatment schedule was used: the vehicle control arm, 100 μl of 0.9% saline administered intraperitoneally on days 1, 2, and 3; the LY2157299 arm, 4 mg/kg administered intraperitoneally on days 1, 2, and 3; the drug treatment arm, cisplatin (2.5 mg/kg) administered intraperitoneally on day 1 plus etoposide (4 mg/kg) administered intraperitoneally on days 1, 2, and 3; and the combination arm, LY2157299 (2 mg/kg) administered intraperitoneally on days 1, 2, and 3 plus cisplatin (1.5 mg/kg) administered intraperitoneally on day 1 and etoposide (3 mg/kg) administered intraperitoneally on days 1, 2, and 3. Animals were given a two-week respite from treatment to allow for weight recovery.

#### Statistics and reproducibility

All experiments were performed independently three or more times under similar conditions. The results are presented as the means, and the error bars represent the standard deviation (SD) unless stated otherwise. Prism 5.0 was used to generate graphs, and SPSS 20.0 was used to perform statistical analyses, with *p* values calculated using the chi-square test, one-way ANOVA or unpaired two-tailed Student’s *t* test. Survival durations were analysed using the Kaplan–Meier method and were compared among groups using the log-rank test. Hazard ratios were calculated using a Cox-regression analysis. Significant *p* values are represented as *p ≤ 0.05, **p ≤ 0.01, ***p < 0.001, and n.s. no significance.

## Results

### Deregulated circRNAs in chemoresistant SCLC and cESRP1 characterisation

**Fig. 1:**
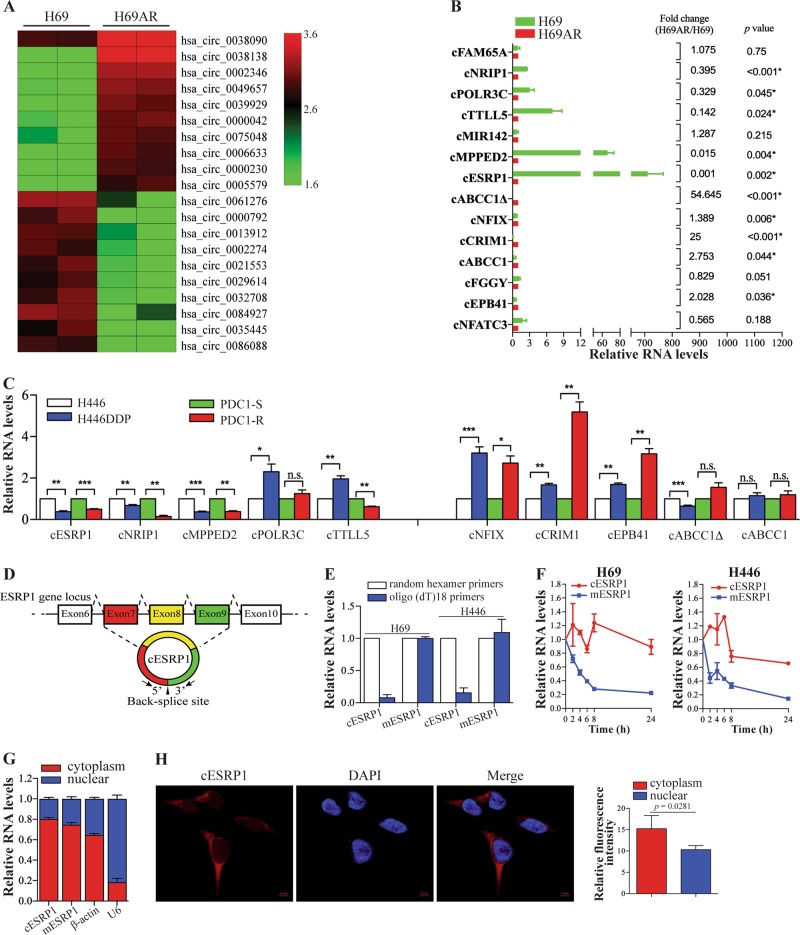
Deregulated circRNAs in chemoresistant SCLC and cESRP1 characterisation. **a** The heatmap shows the top ten circRNAs with the most increased and decreased expression in H69AR cells compared with H69 cells, as determined using a circRNA Arraystar Chip. **b** We validated the differential expression of 14 circRNAs in H69AR cells and H69 cells using qRT-PCR. An independent-sample *t*-test was used; qRT-PCR, quantitative reverse transcription PCR. Data are mean ± SD, *n* = 3. **c** The relative expression of the ten indicated circRNAs listed in **a** from the chemoresistant cells and matched chemosensitive cells was measured by qRT-PCR; PDC1-S, patient-derived cells that are relatively sensitive to chemotherapy; PDC1-R, patient-derived cells that are relatively resistant to chemotherapy. Data are mean ± SD, *n* = 3. **d** A schematic diagram of the genomic location and splicing pattern of cESRP1 is shown. **e** Random hexamer or oligo (dT)18 primers were used in reverse transcription experiments. The relative RNA levels were analysed by qRT-PCR and normalised to the level measured using the random hexamer primers. Data are mean ± SD, *n* = 3. **f** The relative RNA levels of cESRP1 and mESRP1 in H69 and H446 cells were analysed by qRT-PCR after treatment with actinomycin D at the indicated time points. Data are mean ± SD, *n* = 3. **g** cESRP1 and mESRP1 were abundant in the cytoplasm of H69 cells. β-actin and U6 were used as positive controls in the cytoplasm and nucleus, respectively. Data are mean ± SD, *n* = 3. **h** RNA fluorescence in situ hybridisation for cESRP1 was performed in H446 cells. Nuclei were stained with 4,6-diamidino-2-phenylindole (DAPI). Scale bar, 5 μm

We first analysed the circRNA expression profiles of SCLC cells by using a circRNA microarray (Fig. S1a; Supplementary Table 1). The expression analysis results identified 224 differentially expressed circRNAs, with 138 circRNAs exhibiting upregulated expression and 86 exhibiting downregulated expression in H69AR as compared with H69. Ten circRNAs with the greatest increases and decreases in expression are shown in Fig. 1a.

We verified part of circRNA microarray results by qRT-PCR in H69AR/H69 cells as well as in the other two compared cells H446DDP/H446 and PDC1-R/PDC1-S. We validated five upregulated and five downregulated circRNAs observed in H69AR cells using circRNA-specific divergent primers and Sanger sequencing (Fig. 1b; Fig. S1b). Furthermore, these ten circRNAs were proven to be real circRNAs after treatment with RNase R (Fig. S1c). Of the ten circRNAs, we observed six circRNAs (cESRP1, hsa_circ_0084927; cNRIP1, hsa_circ_0061276; cMPPED2, hsa_circ_0021553; cNFIX, hsa_circ_0049657; cCRIM1, hsa_circ_0005579; and cEPB41, hsa_circ_0000042) with consistent and significant deregulated expression in chemorensistant cells (Fig. 1c). Among these circRNAs, because cESRP1 originates from the ESRP1 gene, which is closely linked to tumour-associated EMT [45, 46], we selected cESRP1 to further investigate the role of cESRP1 in SCLC chemoresistance.

cESRP1 is produced from exons 7, 8, and 9 of the ESRP1 gene locus (CircBase ID: hsa_circ_0084927, referred to as cESRP1, http://www.circbase.org/cgi-bin/listsearch.cgi) (Fig. 1d). To confirm the characteristics of cESRP1, we used random hexamer or oligo (dT)18 primers in reverse transcription experiments. Compared with the results obtained using the random hexamer primers, the relative expression of cESRP1 was observed to be significantly downregulated using the oligo (dT)18 primers, whereas the linear RNA mESRP1 RNA was not (Fig. 1e). This finding suggested that cESRP1 did not have a poly-A tail. Furthermore, actinomycin D was used to inhibit transcription and compare the half-lives of cESRP1 and mESRP1, the results of which showed that cESRP1 was more stable than mESRP1 in SCLC cells (Fig. 1f). We also presented cESRP1 was primarily localised in the cytoplasm in SCLC cells by qRT-PCR and FISH (Fig. 1g, h).

### cESRP1 inhibits SCLC chemoresistance in vitro and in vivo

**Fig. 2:**
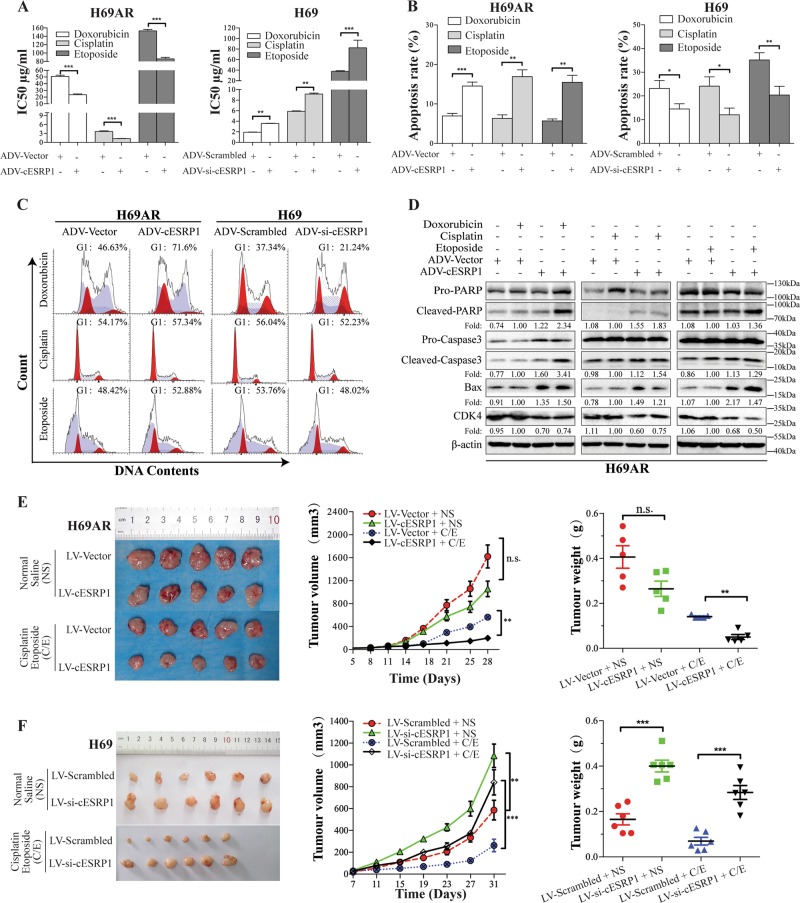
cESRP1 inhibits SCLC chemoresistance. **a** Bar graph representing the effect of cESRP1 modulation on the drug sensitivity of SCLC cells measured by a CCK8 assay. Data were pooled from four biological replicates ± SD, *n* = 4. **b** Bar graph showing the effect of cESRP1 modulation on the cell apoptosis rate after chemotherapy treatment in SCLC cells. Data are mean ± SD, *n* = 3. **c** FACS analysis showing an increase in the proportion of cells in the G1 phase in chemoresistant cells overexpressing cESRP1 and treated with chemotherapeutic drugs, whereas a decrease in the G1 population was observed when cESRP1 was silenced in chemosensitive cells. **d** PARP, caspase3, Bax, and CDK4 expression levels detected by western blot analysis in cESRP1-overexpressing H69AR cells following treatment with chemotherapeutic drugs. **e** Effects of cESRP1 overexpression on tumour growth in vivo. Left: Representative images of tumours formed in nude mice after subcutaneous injection of cESRP1-overexpressing H69AR cells are shown (*n* = 5). Middle: Tumours comprising cESRP1-overexpressing H69AR cells showed markedly lower growth rates than those comprising control cells. Right: Tumour weight was measured. Data are mean ± SD. **f** Effects of cESRP1 expression knockdown on tumour growth in vivo. Left: Representative images of tumours formed in nude mice from subcutaneously injected cESRP1-knockdown H69 cells (*n* = 6). Middle: Tumour growth curves. Tumours comprising cESRP1-knockdown cells showed markedly higher growth rates than control cells. Right: Tumour weight was measured. Data are mean ± SD

We further investigated the function of cESRP1 in SCLC chemoresistance by first constructing transient cESRP1-overexpressing cells (ADV-cESRP1) and cESRP1-knockdown cells (ADV-si-cESRP1) using adenoviral vectors in chemoresistant and chemosensitive cells, respectively (Fig. S2a). Subsequently, cell viability, cell cycle distribution, and cell apoptosis were examined after treatment with chemotherapeutic drugs, including doxorubicin, cisplatin, and etoposide. The results indicated that cESRP1 upregulation promoted cell chemosensitivity, with a significant decrease in the IC50 value observed as well as increases in cell apoptosis and G0/G1 cell arrest, whereas reduced cESRP1 expression led to the opposite results (Fig. 2a–c; Fig. S2b–d). Immunoblotting was performed to assess proteins levels in apoptotic and cell cycle pathways. Notably, the immunoblotting results showed that apoptotic protein markers, including cleaved PARP, cleaved caspase3, and Bax, increased after cESRP1 overexpression in chemoresistant cells. We also observed that the level of the G1/S phase checkpoint protein CDK4 decreased after treatment with doxorubicin, cisplatin, or etoposide in the cESRP1-overexpressing cells compared with the vector-transfected cells (Fig. 2d; Fig. S2e).

We next tested the effect of cESRP1 on tumour chemotherapeutic reactivity in vivo. We constructed cell lines stably overexpressing or underexpressing cESRP1 (H69AR-LV-cESRP1 and H69-LV-si-cESRP1, respectively) using lentiviral vectors. We then performed a tumourigenesis assay by subcutaneously injecting cESRP1-overexpressing cells or control cells into the flanks of nude mice. The results showed that tumour growth was slowed by cESRP1 overexpression in both the normal saline (NS) cohort and the C/E-treated cohort (Fig. 2e; Fig. S2f). In contrast, we observed that tumour growth was accelerated by cESRP1 knockdown (Fig. 2f). Together, these data indicated that cESRP1 rescues SCLC chemoresistance.

### cESRP1 may function as a sponge of miR-93-5p in SCLC

**Fig. 3:**
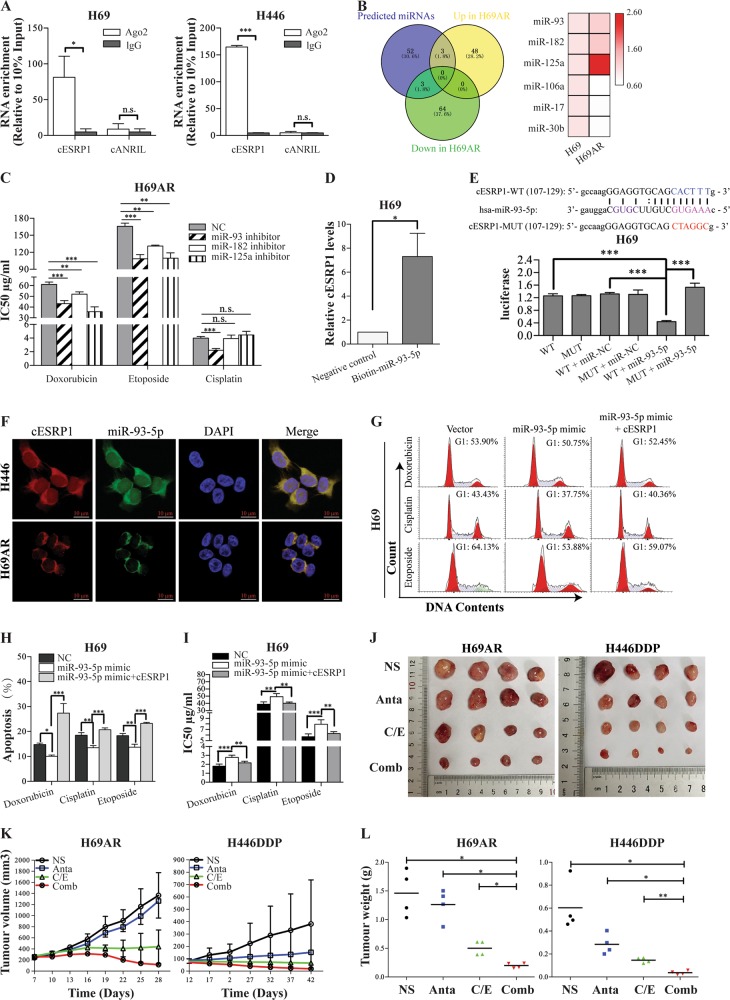
cESRP1 functions as a sponge of miR-93-5p in SCLC. **a** RIP experiments were performed using an antibody against AGO2 with extracts from SCLC cells. Data are mean ± SD, *n* = 3. **b** The Venn diagram shows the intersection of miRNA lists. The heatmap shows the expression of the overlapping miRNAs in chemoresistant and chemosensitive SCLC cell lines. **c** The bar graph shows the effect of miR-93-5p, miR-182-5p, and miR-125a-5p inhibition on the drug sensitivity of H69AR cells. Data were pooled from four biological replicates ± SD, *n* = 4. **d** qRT-PCR was used to analyse the cESRP1 levels in streptavidin-captured fractions from H69 cell lysates after transfection with 3′-end biotinylated miR-93-5p or a negative control. Data are mean ± SD, *n* = 3. **e** H69 cells were co-transfected with LUC-cESRP1-WT or LUC-cESRP1-MUT vectors and an miR-93-5p mimic or a negative control (miR-NC). Luciferase activity was detected with luciferase reporter assays. Data are mean ± SD, *n* = 3. **f** The colocalization of cESRP1 and miR-93-5p was observed by RNA in situ hybridisation in H446 and H69AR cells. Nuclei were stained with a DAPI solution. **g**–**h** Cell cycle and cell apoptosis rate analyses of H69 cells that received the indicated treatments are shown. Data are mean ± SD, *n* = 3. **i** The IC50 values of H69 cells transfected with the indicated transcripts and treated with drugs were measured using CCK-8 assays. Data are mean ± SD, *n* = 4. **j** Images of subcutaneous tumours comprising H69AR or H446DDP cells after cisplatin/etoposide (C/E) treatment or combination treatment (Comb) with the miR-93-5p antagomir (Anta) are shown (*n* = 4). NS normal saline. **k** The growth curves of xenografted tumours derived from SCLC cells with or without miR-93-5p antagomir or cisplatin/etoposide treatment are shown. Data are mean ± SD, *n* = 4. **l** Tumour weight (means) was measured at the endpoint

It is accepted that circRNAs can function as sponges of miRNAs and that cESRP1 is stable and located in the cytoplasm of SCLC cells; thus, exploring whether cESRP1 can bind to miRNAs may be valuable. We first conducted an RIP assay with an antibody against AGO2 in SCLC cells. The results showed that cESRP1 but not the negative control cANRIL [47], was significantly enriched using the anti-AGO2 antibody (Fig. 3a). This result suggested that cESRP1 may act as an miRNA sponge to be involved in the AGO2-mediated ceRNA mechanism of action.

We next examined potential miRNAs associated with cESRP1 using the miRcode and TargetScan prediction tools (Supplementary Table 2). According to our miRNA microarray [48] and the above-predicted miRNAs, three miRNAs (miR-93, miR-182, miR-125a) were up-expressed, and three (miR-106a, miR-17, miR-30b) down-expressed in H69AR cells compared with H69 cells (Fig. 3b; Supplementary Table 3). However, the expression of these six miRNAs was upregulated in chemoresistant cells compared with chemosensitive cells, as confirmed by qRT-PCR assays (Fig. S3a). Subsequently, we observed that the enrichment of these six miRNAs was unaffected by the cESRP1 levels in SCLC cells (Fig. S3b). These findings suggest that miRNAs may not be degraded by cESRP1, as previously described [47]. Based on the results of previous studies [49–52], we focused on miR-93-5p, miR-182-5p, and miR-125a-5p and further investigated their roles in chemoresistance in SCLC cells. Using specific miRNA inhibitors, we successfully downregulated the expression of these three miRNAs in SCLC cells (Fig. S3c) and then treated the cells with chemotherapeutic drugs. We observed that the inhibition of miR-182-5p, miR-93-5p, and miR-125a-5p promoted chemosensitivity to doxorubicin and etoposide. In addition, miR-93-5p knockdown also facilitated chemosensitivity to cisplatin (Fig. 3c). Thus, we selected miR-93-5p for the follow-up studies. To identify whether miR-93-5p can bind to cESRP1, we purified miR-93-5p-associated RNAs by biotin-miRNA pulldown and observed a 7.3-fold enrichment in cESRP1 in the biotinylated miR-93-5p-captured fraction compared with the negative control fraction (Fig. 3d). To further determine whether cESRP1 harbours an miR-93-5p binding domain, we constructed a luciferase reporter gene vector containing either the WT or mutant (MUT) cESRP1 and then co-transfected miR-93-5p mimics with the luciferase reporter gene vectors into H69 and H446 cells. Compared with a negative control RNA, the miR-93-5p mimic reduced the luciferase reporter activity in SCLC cells transfected with the WT cESRP1 construct (Fig. 3e; Fig. S3d). In addition, double FISH indicated the colocalization of cESRP1 and miR-93-5p in H446 and H69AR cells by fluorescence confocal microscopy (Fig. 3f).

Subsequently, we further assessed the effects of cESRP1 on the function of miR-93-5p with respect to drug resistance in SCLC. Our results showed that miR-93-5p overexpression significantly decreased the G0/G1 cell proportion and cell apoptosis but markedly enhanced cell viability in response to drug treatment, whereas reintroducing exogenous cESRP1 abolished the drug resistance-promoting effects of miR-93-5p on SCLC cells in vitro (Fig. 3g–i; Fig. S3e-f). Notably, intravenous administration of a cholesterol-modified miR-93-5p antagomir markedly enhanced the sensitivity of tumours to chemotherapeutic drugs in vivo (Fig. 3j–l). The results of these experiments indicate that cESRP1 may function as a sponge of miR-93-5p in SCLC.

### cESRP1 prohibits TGF-β-mediated EMT via the miR-93-5p-Smad7/p21(CDKN1A) axis in SCLC

**Fig. 4:**
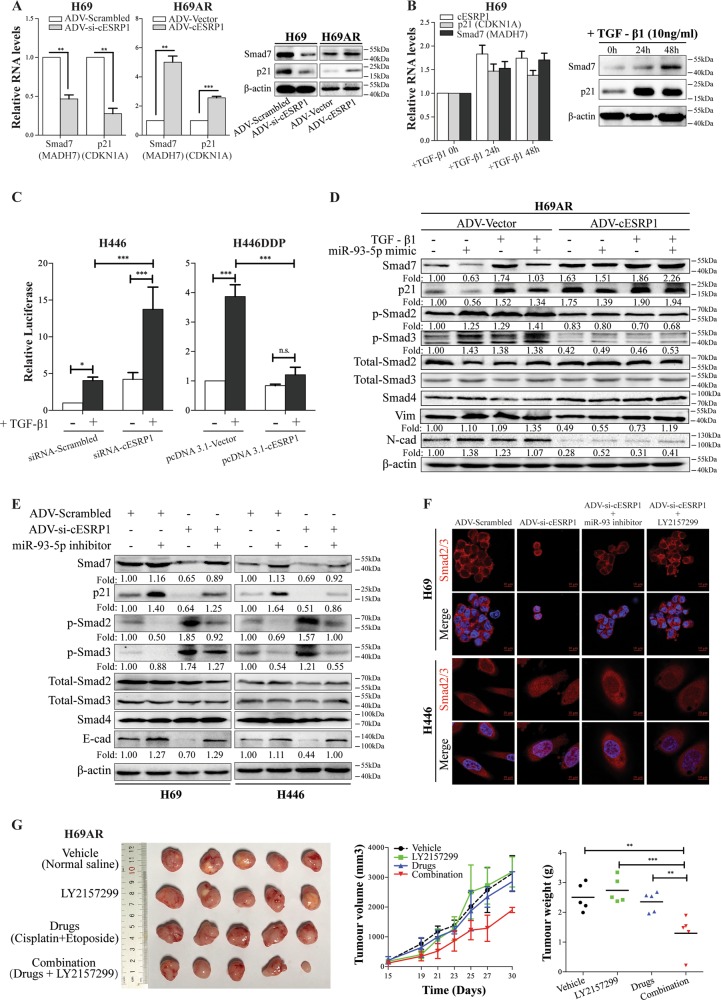
cESRP1 prohibits TGF-β-mediated EMT via the miR-93-5p-Smad7/p21(CDKN1A) axis. **a** Smad7 and p21(CDKN1A) expression were analysed after interfering with cESRP1 expression in SCLC cells. Data are mean ± SD, *n* = 3. **b** The transcriptional or translational levels of cESRP1, Smad7, and p21(CDKN1A) were upregulated after treatment of H69 cells with TGF-β1. Data are mean ± SD, *n* = 3. **c** SCLC cells were co-transfected with a CAGA-luciferase reporter and cESRP1-siRNA vector or cESRP1-overexpressing vector. The cells were stimulated with TGF-β1 overnight, and luciferase activity was measured. A scrambled vector or an empty vector was used as a control. The data are presented as the means of triplicate samples from a representative experiment performed three times ± SD. **d** Western blotting was used to analyse cESRP1-overexpressing H69AR cells treated with TGF-β1 (10 ng/ml) or transiently transfected with an miR-93-5p mimic. **e** Western blot analysis of SCLC chemosensitive cells transiently transfected with a miR-93-5p inhibitor after the transient knockdown of cESRP1 expression by an adenovirus. **f** Immunofluorescence staining shows intracellular Smad2/3 in the indicated cells. **g** A subcutaneous implantation model was established using H69AR cells. The graphs below show the tumours that developed and a statistical plot of the tumour growth kinetics (means ± SD) as well as the average tumour weights in the subcutaneous implantation model mice. *n* = 5

Previous reports have demonstrated that the miR-106b-25 cluster (miR-106b, miR-93, and miR-25), which is activated by E2F1, can inhibit the growth-suppressing functions of TGF-β signalling by interfering with the downstream mediators p21 (CDKN1A, Waf1/Cip1) and Bim (BCL2L11) in gastric cancer [53]. These miRNAs can also target the TGF-β inhibitor Smad7 to activate the TGF-β signalling and induce EMT and tumour initiating cell characteristics downstream of Six1 in human breast cancer [54]. Based on these findings, we hypothesised that cESRP1 inhibits chemoresistance in SCLC by protecting Smad7 and p21(CDKN1A) from downregulation by miR-93-5p, thus inhibiting the tumour-promoting functions of TGF-β signalling. To test this hypothesis, we first examined the expression of Smad7 and p21(CDKN1A) in chemosensitive and chemoresistant SCLC cells, both of which were observed to be significantly downregulated in the chemoresistant cells (Fig. S4a). Second, we found the Smad7 and p21(CDKN1A) levels were significantly downregulated upon miR-93-5p overexpression, whereas they were significantly upregulated when miR-93-5p knockdown in SCLC cells (Fig. S4b). Third, we demonstrated that the Smad7/p21(CDKN1A) levels were strikingly upregulated when cESRP1 was overexpressed and downregulated when cESRP1 expression was knocked down (Fig. 4a; Fig. S4c). More interestingly, with the increase in exogenous cESRP1 expression, the expression of Smad7/p21(CDKN1A) was less inhibited by the exogenous miR-93-5p mimic (Fig. S4d). These data indicated that miR-93-5p and cESRP1 efficiently interfered with Smad7/p21(CDKN1A) expression in SCLC.

Many studies have shown that TGF-β can serve as a tumour suppressors to induce the expression of Smad7 and p21(CDKN1A) in certain cancers [55–57]. Interestingly, Smad7 and p21(CDKN1A) may act as components in a negative feedback regulation of TGF-β signalling, as Smad7 impedes TGF-β/Smad-driven transcription [21, 58, 59]. Although p21(CDKN1A) is a direct target of Smad2/3 [60], it could repress features of EMT by non-canonical TGF-β pathways such as c-Myc and TGFβ/MEK/ERK signalling [31, 32]. To investigate whether cESRP1 is also involved in the negative regulation of TGF-β signalling in SCLC, we first treated chemosensitive cells with TGF-β1 (Fig. S4e) and observed that the TGF-β1 treatment efficiently increased the expression of cESRP1, Smad7, and p21(CDKN1A) (Fig. 4b; Fig. S4f). These results are consistent with those of previous studies [18, 61, 62]. Furthermore, our data revealed that the upregulation of cESRP1 by TGF-β1 is independent of changes in the parental mESRP1 transcript (Fig. S4g). We then asked whether cESRP1 is a relevant factor in the regulation of TGF-β activity in SCLC. We observed that silencing cESRP1 greatly enhanced the CAGA-luciferase activity of a TGF-β-responsive reporter, whereas ectopic expression of cESRP1 weakened CAGA-luciferase activity (Fig. 4c). Furthermore, the transient overexpression of cESRP1 resulted in a decrease in the protein levels of p-Smad2/3 and the mesenchymal markers N-cadherin and vimentin, while treatment with a miR-93-5p mimic could partly block cESRP1-induced TGF-β/Smad signalling inhibition (Fig. 4d; Fig. S4h). In contrast, cESRP1 silencing enhanced TGF-β-Smad2/3 pathway activity, as evidenced by increased levels of p-Smad2/3 and decreased expression of the epithelial marker E-cadherin, whereas downregulating miR-93-5p expression reversed the effect of cESRP1-siRNA (Fig. 4e). Importantly, Smad2/3 predominantly accumulated in the nucleus of cESRP1-silenced cells, while knocking down miR-93-5p expression or treating cells with the TGF-β signalling inhibitor LY2157299 reversed the nuclear translocation of Smads induced by cESRP1 silencing (Fig. 4f). The miR-93-5p mimic and TGF-β1 could both rescue the cESRP1-mediated effect on the cytoplasmic localisation of Smad2/3 in the SCLC cells (Fig. S4i).

To further investigate the functional links between TGF-β signalling and drug resistance in vivo, we treated SCLC chemoresistant cell-derived xenograft mice with either vehicle or the TGF-β signalling inhibitor LY2157299 in conjunction with chemotherapy drugs (C/E). Vehicle-treated tumours and LY2157299-treated tumours expanded quickly, whereas a significant decline in tumour growth was observed when the chemotherapy drugs C/E were combined with a TGF-β inhibitor (Fig. 4g). These data underscore that TGF-β inhibition significantly enhances the responsiveness to treatment in SCLC.

### TGF-β signalling inhibition augments the chemoresponsiveness of SCLC patient-derived xenografts

**Fig. 5:**
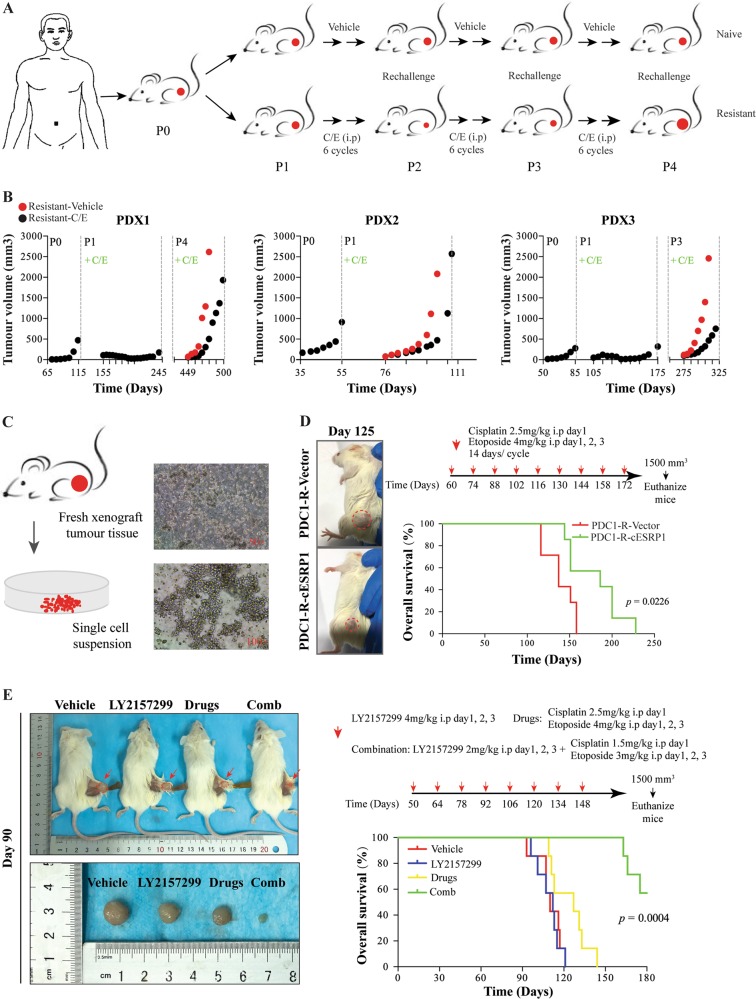
TGF-β signalling inhibition augments the chemoresponsiveness of SCLC PDX. **a** Outline of the experimental approach, beginning with the primary sample. **b** Tumour growth kinetics of PDX1, PDX2, and PDX3 models. Average tumour volumes (*n* = 10) of different generations of xenograft tumours are shown (P0 was the initial xenograft tumour and was not exposed to C/E; P1, P2, P3, and P4 represent the second, third, fourth, and fifth generation re-engrafted tumours, which were treated with C/E (black dots) or a vehicle (red dots)). The ticks on the *x*-axis indicate day 1 of every C/E cycle. The dashed vertical lines and *x*-axis days indicate the times at which the treated tumours were collected and re-engrafted into the next cohort. **c** Digestion of xenograft tumour tissue into single-cell suspensions for in vitro culture. **d** Photograph of mouse tumours on day 125 after implantation of PDC1-R-Vector cells or PDC1-R-cESRP1 cells treated with chemotherapeutic drugs (Left). The overall survival (OS) of the mice in the two indicated groups is shown (Right) (*n* = 7). OS: Time until the tumour volume reached 1500 mm^3^. **e** Representative image of tumours derived from mice on day 90 after re-engraftment of PDX1-P4-resistant tumour tissue (Left) and the OS of the experimental PDX model mice receiving the indicated treatments shown (Right) (*n* = 7). The TGF-β signalling inhibitor LY2157299, when administered in combination with chemotherapeutic drugs, exhibited remarkable inhibitory effects on tumour growth, whereas LY2157299 treatment alone did not display a therapeutic impact

We next utilised SCLC PDX to further understand the effects of TGF-β signalling on SCLC. We treated tumour-bearing mice with repeated chemotherapy cycles that mimicked clinical practice in three independent SCLC PDX models (Fig. 5a; Supplementary Table 4). Histological comparison of the founder (P0) PDX tumours showed strong similarities to corresponding patient tumour samples (Fig. S5a). With multiple cycles of chemotherapy, we observed that PDX1 developed chemoresistance in the fourth round of C/E treatment and that PDX3 acquired chemoresistance in the third round, while the PDX2 model showed initial chemoresistance without any dose-limiting toxicity as measured by animal weight (Fig. 5b; Fig. S5b).

We established one cell line from fresh drug-resistant PDX1 (PDC1-R) xenograft tumour tissue as described (Fig. 5c). We then stably overexpressed cESRP1 in the PDC1-R cells (Fig. S5c). A subcutaneous xenograft model established with the PDC1-R cells showed that the overall survival (OS) of mice bearing a tumour comprising cESRP1-overexpressing cells was significantly better than that of mice bearing the corresponding control tumour that developed from empty vector-infected PDC1-R cells (Fig. 5d). We also further verified that TGF-β inhibitor significantly enhanced the tumour responsiveness to treatment in SCLC PDX (Fig. 5e).

### cESRP1 expression is downregulated in SCLC tissues and predicts a poor prognosis

**Fig. 6:**
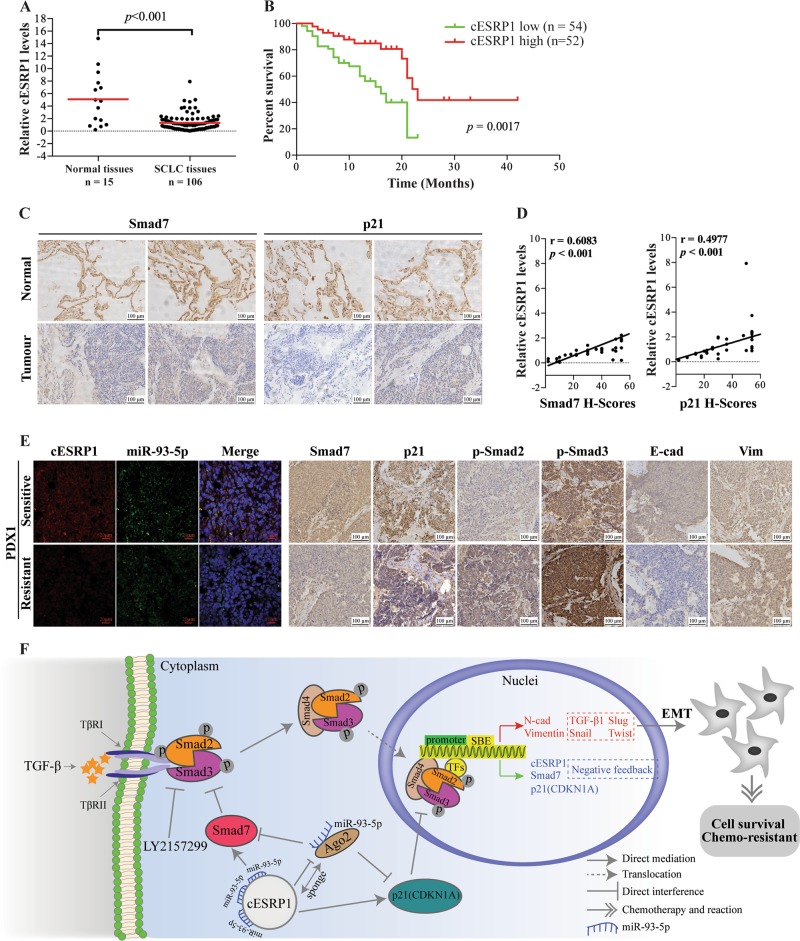
cESRP1 expression is downregulated in SCLC tissues and low expression of cESRP1 predicts a poor prognosis. **a** The differential expression of cESRP1 between SCLC tissue samples and matched normal tissue samples was assessed. The median cESRP1 expression level of each group is indicated by a horizontal line in the scatterplot. **b** Kaplan–Meier survival curves show the correlations between cESRP1 expression and overall survival in SCLC patients. **c** Immunohistochemical staining for Smad7/p21 in SCLC tissue samples and adjacent normal lung tissue samples is shown. **d** The expression levels of Smad7/p21 positively correlated with those of cESRP1 in SCLC tissue samples (*n* = 48). **e** cESRP1 expression was assessed in situ by an RNA FISH assay, and Smad7/p21/p-Smad2/p-Smad3/E-cad/vimentin expression was assessed by IHC in paraffin-embedded sections of PDX1 tissue samples. **f** A schematic drawing indicates the mechanism by which cESRP1 upregulates Smad7/p21(CDKN1A) expression to inactivate the TGF-β/Smad pathway and inhibit EMT to enhance the chemosensitivity of SCLC

We further analysed whether decreased cESRP1 expression correlates with SCLC patient prognosis. As shown in Fig. 6a, cESRP1 expression was significantly decreased in 106 SCLC patient tissues compared with matched non-tumour tissue samples. The results showed that a lower level of cESRP1 expression significantly correlated with extensive disease-SCLC (ED-SCLC) and a worse status (Table 1). Furthermore, Kaplan–Meier survival curves showed that the patients with SCLC and lower cESRP1 expression had poorer OS than the patients with SCLC and higher cESRP1 expression (Fig. 6b).

**Table 1:** Clinical characteristics of 106 patients with SCLC according to the cESRP1 expression level

Variable	cESRP1		*p* value
	Low	High	
Age, year, ≤62: >62	24:30	30:22	0.173
Sex, male:female	44:10	42:9	0.908
Smocking history, Yes:No	29:23	24:22	0.722
Disease stage, LD:ED	15:37	29:17	0.001*
Status, Survival:Death	30:24	40:12	0.020*

χ^2^ test was used to test the association between two categorical variables (*represents statistically significant differences (*p* < 0.05))

*LD* limited-stage diseases, *ED* extensive-stage disease

A subsequent univariate analysis showed that the cESRP1 expression level and SCLC disease stage significantly correlated with OS in SCLC patients. Moreover, multivariate analysis showed that low expression of cESRP1 could be a factor for predicting poor survival when cESRP1 expression, age, sex, disease stage, and smoking history were included (Table 2). These data indicated that decreased cESRP1 expression is associated with SCLC progression and that the cESRP1 level can be used as an independent prognostic marker in patients with SCLC.

**Table 2:** Univariate and multivariate Cox-regression analysis of various prognostic parameters in patients with SCLC  (*represents statistically significant differences (*p* < 0.05))

Variable	Univariate analysis		Multivariate analysis		
	*p*	HR	*p*	HR (95% CI)	Coefficient
cESRP1	0.010*	0.590	0.046*	0.66 (0.44–0.99)	−0.409
Age	0.724	1.007	0.914	1.00 (0.96–1.92)	0.002
Sex	0.868	1.072	0.546	0.75 (0.29–1.92)	−0.300
Disease stage	<0.001*	4.583	<0.001*	4.33 (1.91–9.78)	1.464
Smoking history	0.478	1.276	0.6	1.22 (0.58–2.57)	0.200

We also observed the significant positive correlations between cESRP1 and the mRNA expression of Smad7/p21(CDKN1A) in the tissue samples by qRT-PCR (Fig. S6a). Subsequently, the IHC results revealed decreased Smad7 and p21(CDKN1A) expression in the SCLC tissue samples and were positively correlated with the cESRP1 levels (Fig. 6c, d).

Furthermore, we found that cESRP1 expression was significantly higher in the chemo-naive tissue samples than that in the paired PDX chemoresistant tissue samples, whereas miR-93-5p abundance was similar between the two sets of samples using RNA FISH assay. We also confirmed that the levels of Smad7/p21 (CDKN1A) and the epithelial marker E-cadherin were downregulated in the PDX-resistant tissue samples, whereas those of TGF-β-EMT activated markers including p-Smad2/3 and vimentin were upregulated in the chemoresistant tissue samples (Fig. 6e; Fig. S6b). Based on these results, we conclude that cESRP1 may regulate Smad7/p21 (CDKN1A) to negatively impact the TGF-β-EMT signalling pathway in SCLC in vivo.

## Discussion

Increasing evidence suggests that circRNAs are involved in a wide range of biological processes, including the development of cancers [63]. A recent study by Li et al. identified an FLI1 exonic circRNAs that has a functional role in the progression of SCLC [64]. However, chemoresistance-associated circRNAs in SCLC have rarely been reported. In our present study, we screened several differentially expressed circRNAs in chemotherapy-resistant cells using circRNA microarray and qRT-PCR analyses. Among these circRNAs, we identified a novel SCLC associated circRNA (cESRP1) that is produced from exons 7, 8, and 9 of the ESRP1 gene locus and is significantly downregulated in drug-resistant cells compared with drug-sensitive cells. We then investigated the function of cESRP1 in SCLC chemoresistance and observed that it can improve chemosensitivity in H69/H69AR and H446/H446DDP cells in vitro and in vivo. Importantly, cESRP1 expression was significantly decreased in SCLC tissues compared with that observed in matched non-tumour tissues. Lower cESRP1 expression was positively correlated with the SCLC extensive stages of patients and poorer survival. Our results suggest, for the first time, that cESRP1 may play a role in SCLC chemoresistance and serve as a valuable prognostic biomarker for patients with SCLC.

Some circRNAs have been well characterised to function as miRNA sponges in tumorigenesis [11, 47, 49, 65–67]. To better understand the potential role of cESRP1 in SCLC chemoresistance, we confirmed that cESRP1 was primarily located in the cytoplasm, which indicated that cESRP1 may function as an miRNA sponge in SCLC. In addition, our previous studies indicated that specific miRNAs are involved in chemoresistance in SCLC [68, 69]. These results prompted us to further explore the interaction between cESRP1 and its associated miRNA in SCLC. We first investigated the presence of cESRP1 seed binding sites for miR-93-5p, miR-182-5p, and miR-125a-5p by combining our previous miRNA microarray results. However, only miR-93-5p was confirmed to mediate multidrug resistance in SCLC. The results of subsequent luciferase assays, a biotinylated miRNA pulldown assay, and rescue experiments further demonstrated that cESRP1 can inhibit chemoresistance in SCLC by adsorbing miR-93-5p. More strikingly, the combined use of an miR-93-5p antagomir and chemotherapeutic drugs could reverse the resistance of tumours to C/E chemotherapy.

In this study, we further showed that cESRP1 may function as a ceRNA to regulate Smad7/p21(CDKN1A) by sponging miR-93-5p to inhibit SCLC chemoresistance. We also demonstrated that cESRP1 prohibits TGF-β-mediated EMT via an miR-93-5p-Smad7/p21(CDKN1A) axis. Smad7/p21(CDKN1A) have been shown to act as components in the negative feedback regulation of TGF-β signalling [21, 31, 32, 58]. Our results suggested that cESRP1 effectively increases the abundance of Smad7/p21(CDKN1A) and formed a double-negative feedback loop to abolish the effect of the TGF-β/Smad signalling pathway. A growing number of clinical studies have shown that anti-TGF-β therapy has an acceptable safety/tolerability profile and exhibits anti-tumour activity in subsets of patients [70–72]. In this study, we used an SCLC chemoresistant cell line orthotopic xenograft model and observed that the TGF-β signalling inhibitor LY2157299 (galunisertib), which was bioavailable, effectively suppressed the TGF-β signalling pathway, augmenting tumour chemotherapy responsiveness in combination with chemotherapeutic drugs.

It is particularly noteworthy that in our study, we established a progressive chemoresistant PDX model that mimics recurrent chemoresistance during the clinical course of SCLC. We further demonstrated that ectopic cESRP1 overexpression significantly improved the OS of mice in the PDX model. Most importantly, LY2157299 effectively strengthened the inhibition of tumour growth by first-line platinum-based chemotherapy in the chemoresistant PDX model.

In summary, in this study, we provided comprehensive evidence that cESRP1 acts as a suppressor of chemoresistance and is a prognostic biomarker for patients with SCLC. cESRP1 enhances drug sensitivity via the miR-93-5p-Smad7/p21(CDKN1A) axis by inhibiting TGF-β-mediated EMT (Fig. 6f). We also demonstrated that inhibition of TGF-β signalling can diminish tumour growth by a combined treatment with the TGF-β signalling inhibitor LY2157299 and chemotherapeutic drugs in SCLC PDX. These findings have significant implications regarding our understanding of the pathogenesis of SCLC multidrug resistance and highlights the importance of investigating the complicated circRNA-miRNA regulatory gene network as well as TGF-β signalling in SCLC progression and treatment efficacy.

## Supplementary information

Supplementary Figure Legends

Supplementary Table Legends

Figure S1

Figure S2

Figure S3

Figure S4(1)

Figure S4(2)

Figure S5

Figure S6

Supplementary Table S1

Supplementary Table S2

Supplementary Table S3

Supplementary Table S4

Supplemental information
