# Supplementary material for: Circular RNA cESRP1 sensitises small cell lung cancer cells to chemotherapy by sponging miR-93-5p to inhibit TGF-β signalling
Source: Cell Death Differ. 2019 Nov 14;27(5):1709–27. doi: 10.1038/s41418-019-0455-x (PMC7206039; doi:10.1038/s41418-019-0455-x)
Supplement: Supplementary file 1 — Supplementary Figure Legends [file 41418_2019_455_MOESM1_ESM.doc]

**Supplementary Figure Legends**

**Supplemental Figure 1. Deregulated circRNAs in chemoresistant SCLC cells.** (**a**) Heat map of circRNA expression profiles in H69 and H69AR cells. (**b**) Schematic representation of PCR primers designed to be divergent for linear mRNAs and convergent for circRNAs; Sanger sequencing showing the back-splicing events of candidate circRNAs. (**c**) qRT-PCR results showing the resistance of circRNAs to RNase R digestion. GAPDH mRNA served as a negative control. Data are mean ± SD, n = 3.

**Supplemental Figure 2. cESRP1 affects the chemoresistance of SCLC.** (**a**) Verification of the transfection efficiency of an adenovirus by qRT-PCR; Empty Vector and Scrambled siRNA were used as negative controls. Data are mean ± SD, n = 3. (**b**) The IC50 of H446DDP or H446 cells transfected with the indicated transcripts and treated with drugs measured using CCK-8 assays (Means ± SD, n = 4). (**c**) Bar graph representing the effect of cESRP1 modulation on cell apoptosis in H446DDP or H446 cells after chemotherapy (Means ± SD, n = 3). (**d**) FACS analysis showing the G1 phase cell proportions of the indicated groups. (**e**) Apoptosis-related proteins and CDK4 detected by Western Blotting after interference with cESRP1 expression following exposure to chemotherapeutic drugs. (**f**) Expression of the proliferation marker ki67 and the apoptosis marker cleaved caspase3 in the indicated tumour tissue samples.

**Supplemental Figure 3. cESRP1 plays a regulatory role in the chemoresistance of SCLC by inhibiting the function of miR-93-5p.** (**a**) The enrichment of the indicated miRNAs in SCLC cells was measured by qRT-PCR and is shown here. Data are mean ± SD, n = 3. (**b**) The levels of the indicated miRNAs were measured after interfering with cESRP1 expression in SCLC cells. Data are mean ± SD, n = 3. (**c**) The expression of indicated miRNAs was analysed after treating with specific miRNA-inhibitor in H69AR cells. Data are mean ± SD, n = 3. (**d**) H446 cells were cotransfected with LUC-cESRP1-WT or LUC-cESRP1-MUT vector and an miR-93-5p mimic or a negative control (miR-NC). Luciferase activity was detected via luciferase reporter assays. Data are mean ± SD, n = 3. (**e, f**) The cell cycle distributions, cell apoptosis rates, and drug IC50 values of H446 cells after receiving the indicated treatments are shown. Data are mean ± SD, n = 3.

**Supplemental Figure 4. cESRP1 inhibits the activation of the TGF-β/Smad signalling pathway.** (**a**) Smad7/p21(CDKN1A) expression was downregulated in chemoresistant cells compared to the corresponding chemosensitive cells. Data are mean ± SD, n = 3. (**b**) Smad7/p21(CDKN1A) expression was analysed after interfering with miR-93-5p expression in SCLC cells. Data are mean ± SD, n = 3. (**c**) Smad7/p21(CDKN1A) expression was analysed after interfering with cESRP1 expression in H446 or H446DDP cells. Data are mean ± SD, n = 3. (**d**) The expression of Smad7/p21(CDKN1A) increases with the increase of exogenous cESRP1 levels exposed to an miR-93-5p mimic. Data are mean ± SD, n = 3. (**e**) The EMT phenotype develops after the treatment of chemosensitive SCLC cells with TGF-β1. (**f**) The cESRP1, Smad7, and p21(CDKN1A) transcriptional and translational levels were upregulated after treating H446 cells with TGF-β1 (10ng/ml). Data are mean ± SD, n = 3. (**g**) Under the treatment of TGF-β1, the expression pattern of cESRP1 is independent of mESRP1. Data are mean ± SD, n = 3. (**h**) Western blotting was used to analyse cESRP1-expressing H446DDP cells after treatment with TGF-β1 (10 ng/ml) or transiently upregulated miR-93-5p. (**i**) Immunofluorescence staining shows intracellular Smad2/3 in the indicated cells. Original magnification, 630×2×.

**Supplemental Figure 5. Characteristics of the PDX model and the overexpression of cESRP1 in PDX primary cells.** (**a**) H&E-stained sections and immunochemically stained sections of primary and P0 xenograft tumour tissues are shown. (**b**) Animal weights were measured during the study. The data are reported as the mean ± SD; n=10/arm. (**c**) cESRP1 was efficiently overexpressed in PDC1-R cells. Data are mean ± SD, n = 3.

**Supplemental Figure 6. Analysis of the expression of cESRP1 and its downstream molecules in clinical specimens and PDX tissue samples.** (**a**) The mRNA expression levels of Smad7/p21(CDKN1A) positively correlated with the cESRP1 expression levels in SCLC tissue samples (n = 106). (**b**) cESRP1 expression was detected in situ by an RNA FISH assay, and Smad7/p21/p-Smad2/p-Smad3/E-cad/vimentin was detected by IHC in paraffin-embedded sections of PDX2 and PDX3 tissue samples.
