# Supplementary material for: Circular RNA cESRP1 sensitises small cell lung cancer cells to chemotherapy by sponging miR-93-5p to inhibit TGF-β signalling
Source: Cell Death Differ. 2019 Nov 14;27(5):1709–27. doi: 10.1038/s41418-019-0455-x (PMC7206039; doi:10.1038/s41418-019-0455-x)
Supplement: Supplementary file 2 — Supplementary Table Legends [file 41418_2019_455_MOESM2_ESM.doc]

**Supplementary Table Legends**

**Supplementary Table S1. Differentially Expressed CircRNAs between H69AR and H69 cells.**

**Supplementary Table S2. Predicting potential miRNAs that bind to cESRP1 by using miRcode and TargetScan program prediction tools.**

**Supplementary Table S3. Significant differential miRNAs between H69 and H69AR cells measured by miRNA microarray (P value < 0.01).**

**Supplementary Table S4. PDX characteristics used throughout this study.**
