# Supplementary figures and images for: Circular RNA cESRP1 sensitises small cell lung cancer cells to chemotherapy by sponging miR-93-5p to inhibit TGF-β signalling
Source: Cell Death Differ. 2019 Nov 14;27(5):1709–27. doi: 10.1038/s41418-019-0455-x (PMC7206039; doi:10.1038/s41418-019-0455-x)

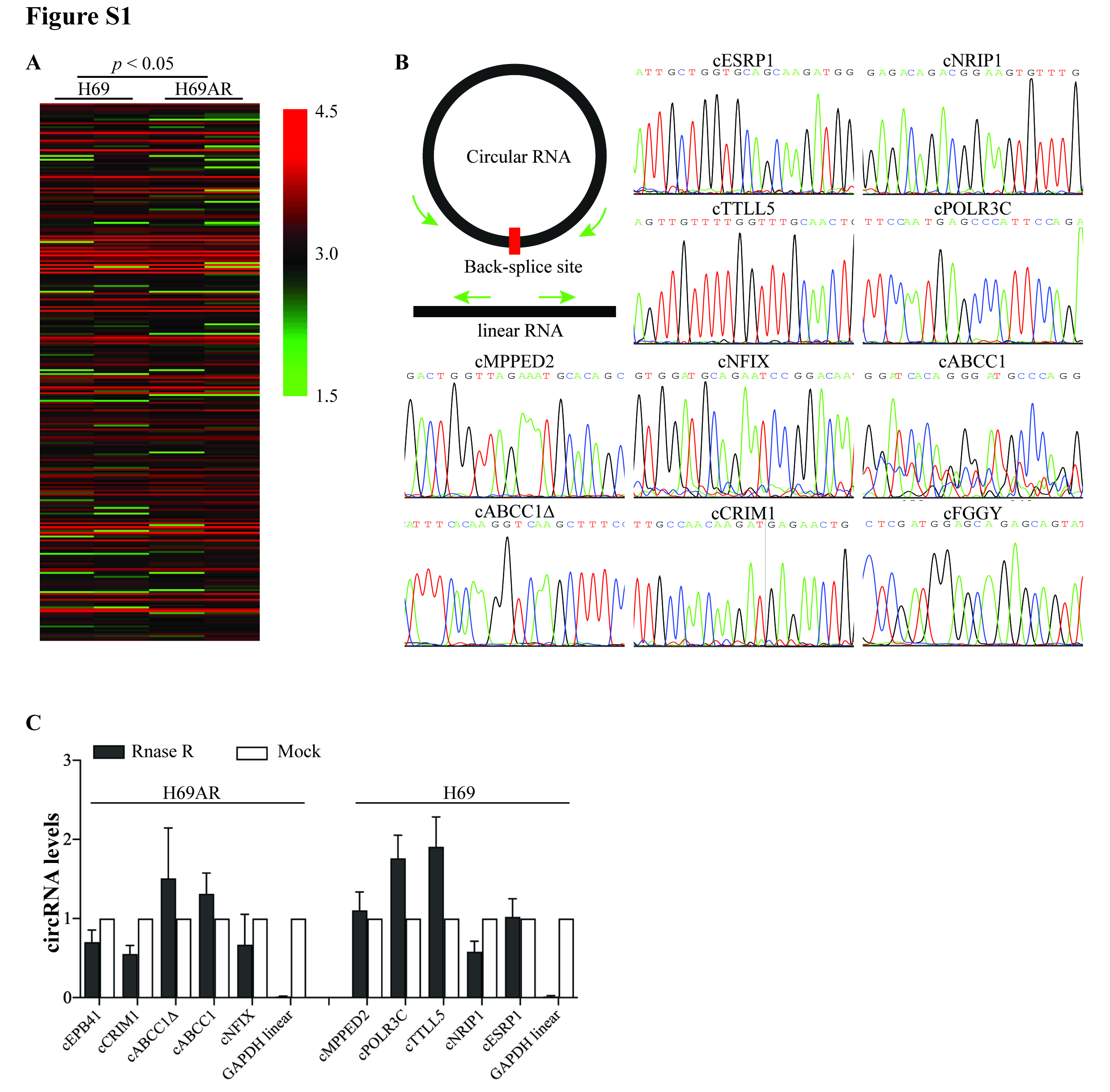

Supplement: Supplementary file 3 — Figure S1 [file 41418_2019_455_MOESM3_ESM.tif]

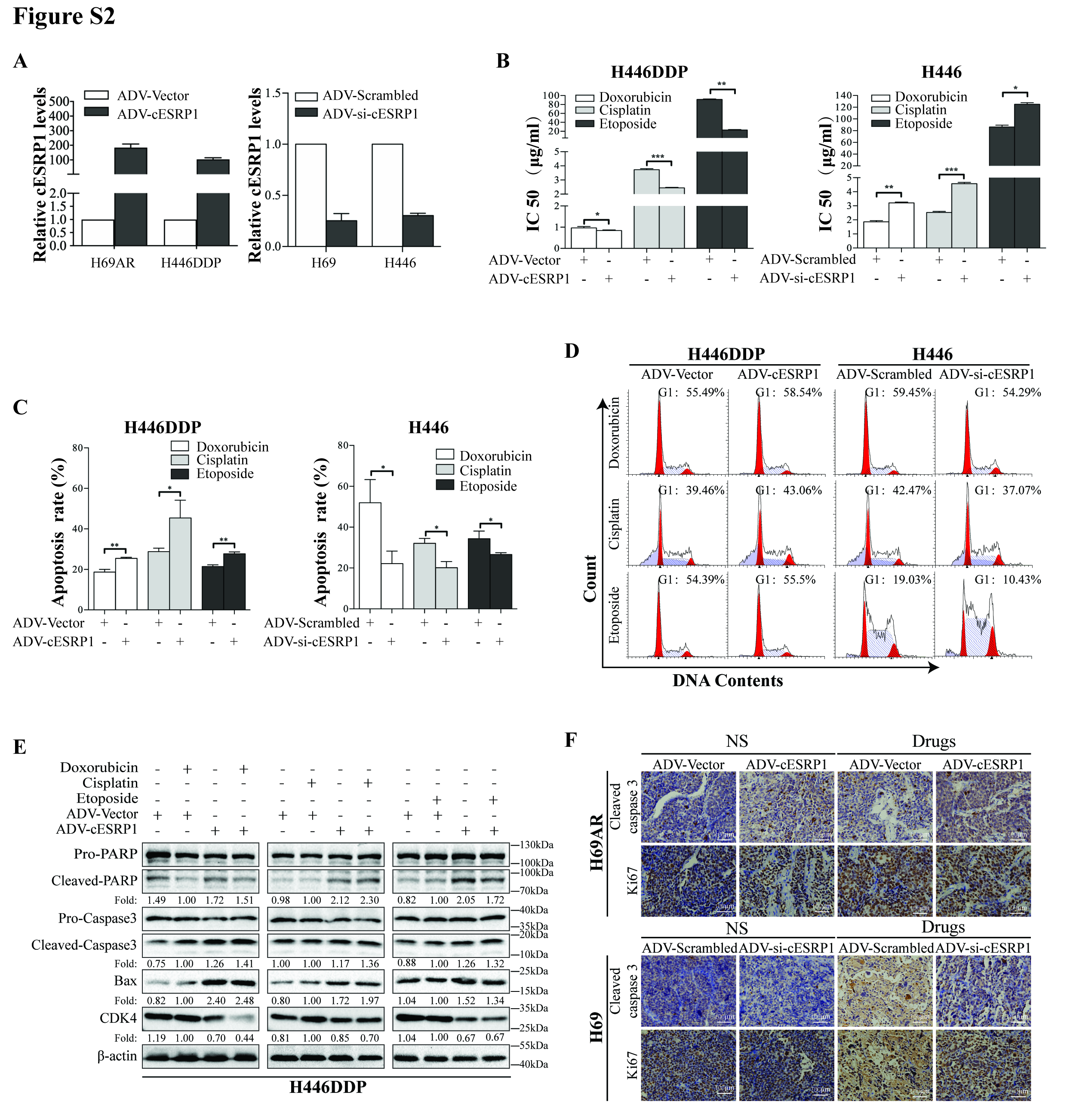

Supplement: Supplementary file 4 — Figure S2 [file 41418_2019_455_MOESM4_ESM.tif]

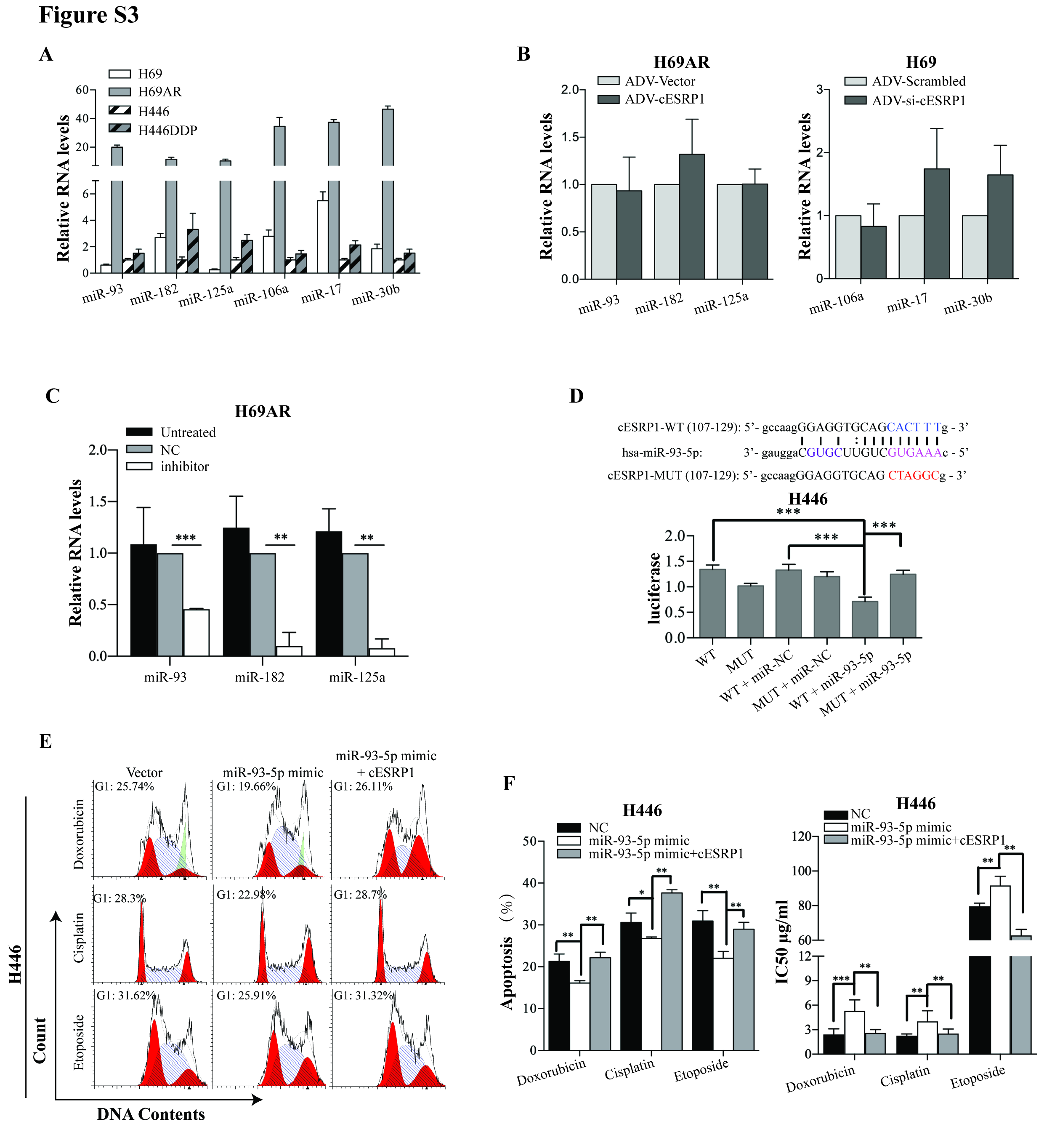

Supplement: Supplementary file 5 — Figure S3 [file 41418_2019_455_MOESM5_ESM.tif]

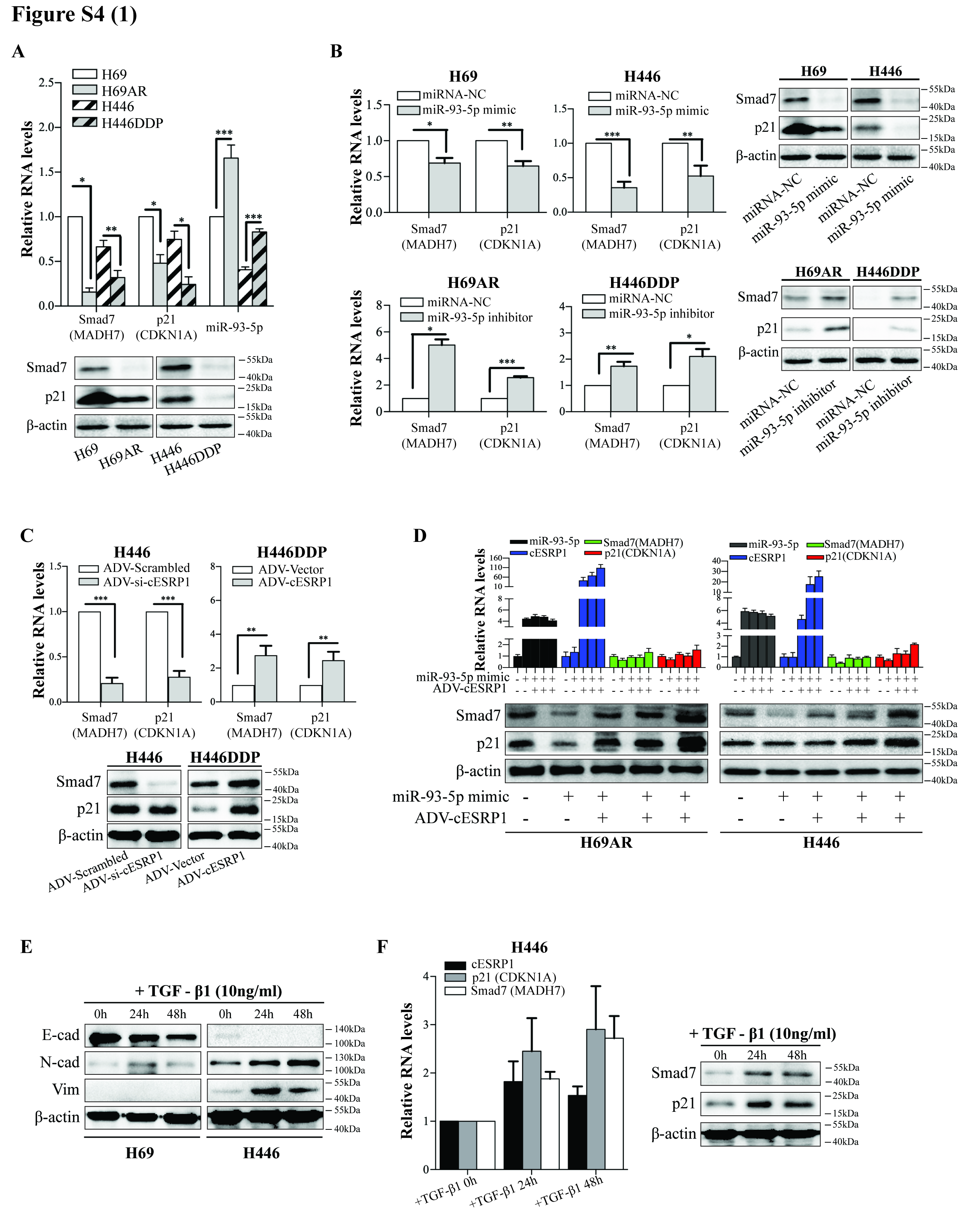

Supplement: Supplementary file 6 — Figure S4(1) [file 41418_2019_455_MOESM6_ESM.tif]

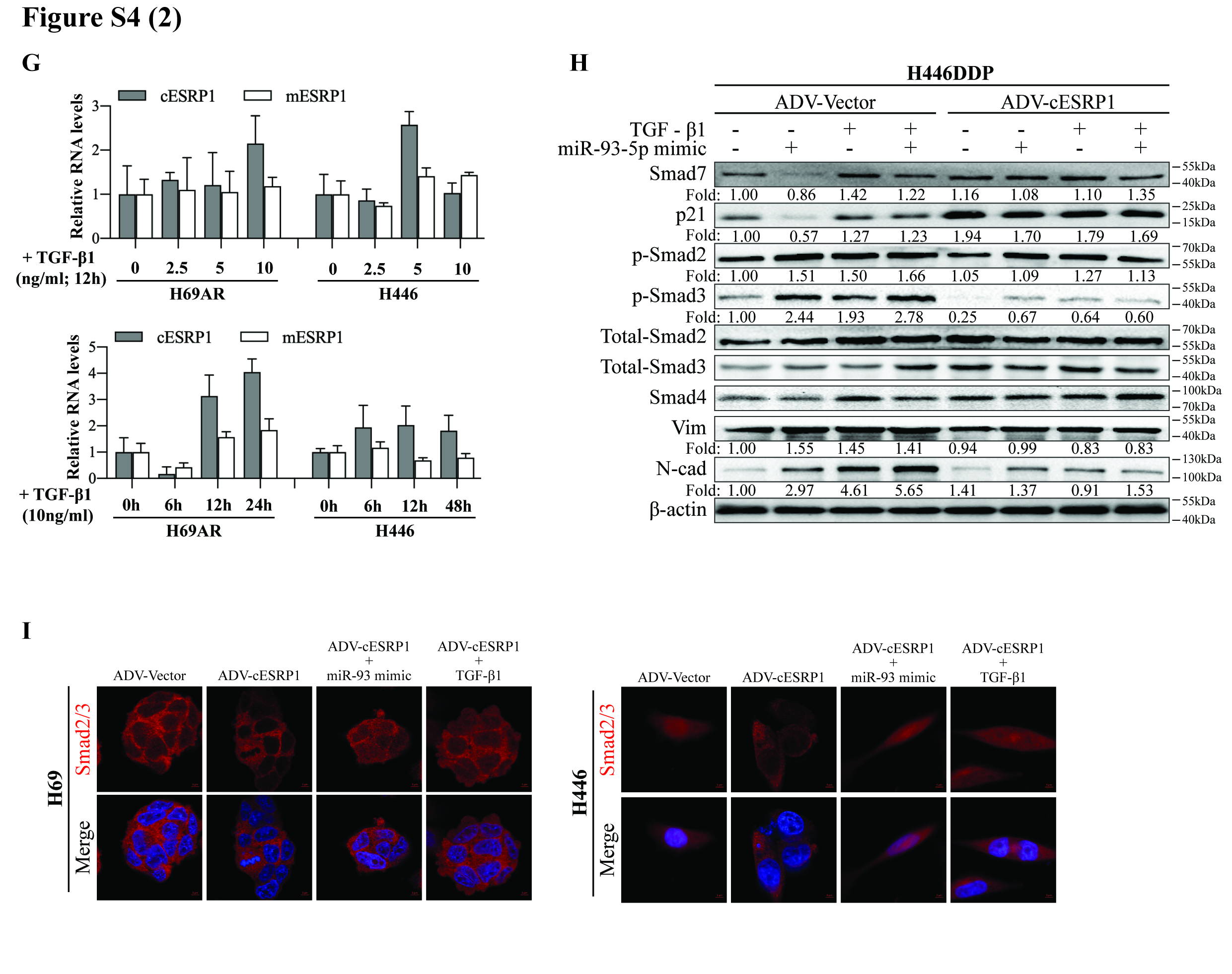

Supplement: Supplementary file 7 — Figure S4(2) [file 41418_2019_455_MOESM7_ESM.tif]

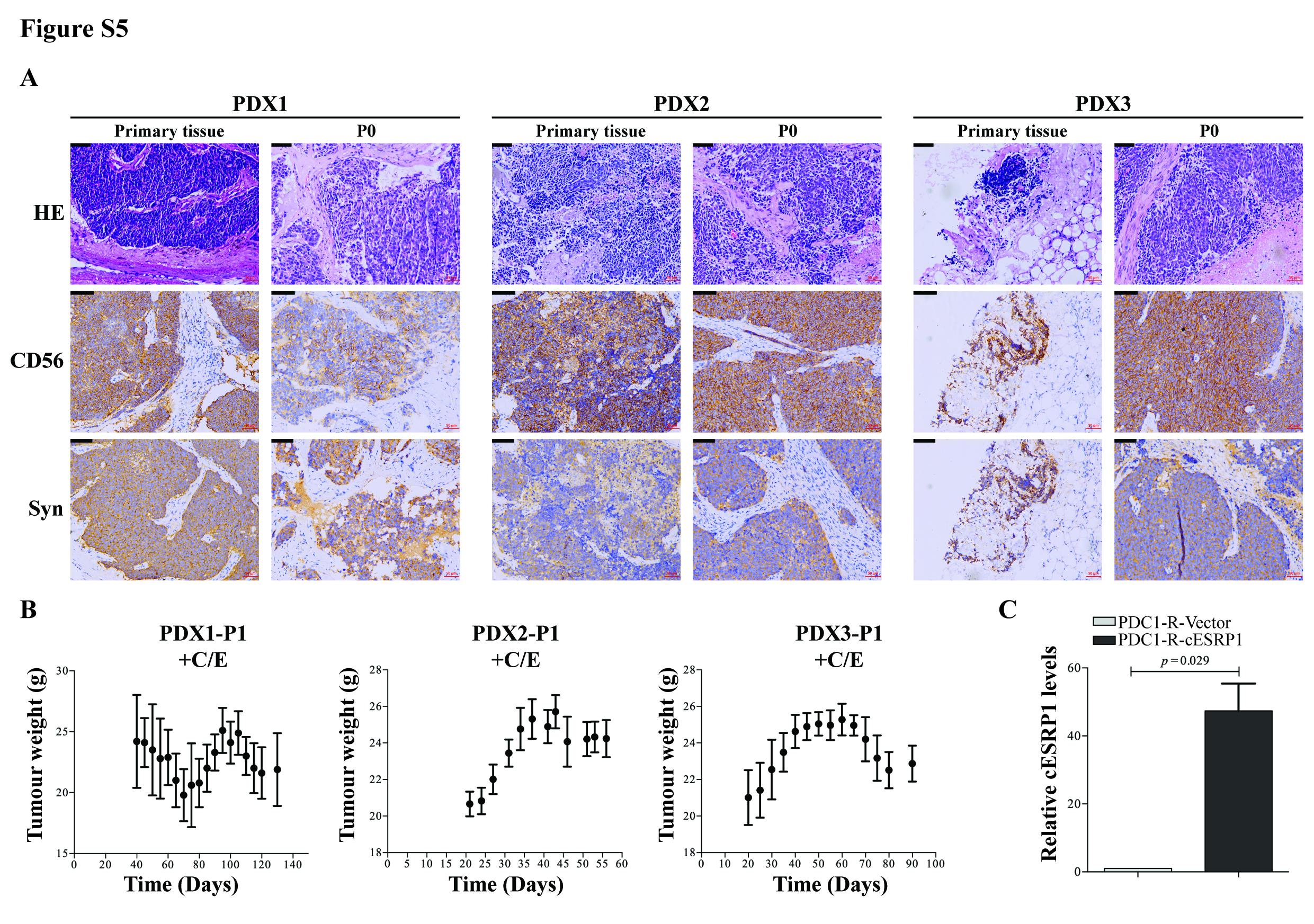

Supplement: Supplementary file 8 — Figure S5 [file 41418_2019_455_MOESM8_ESM.tif]

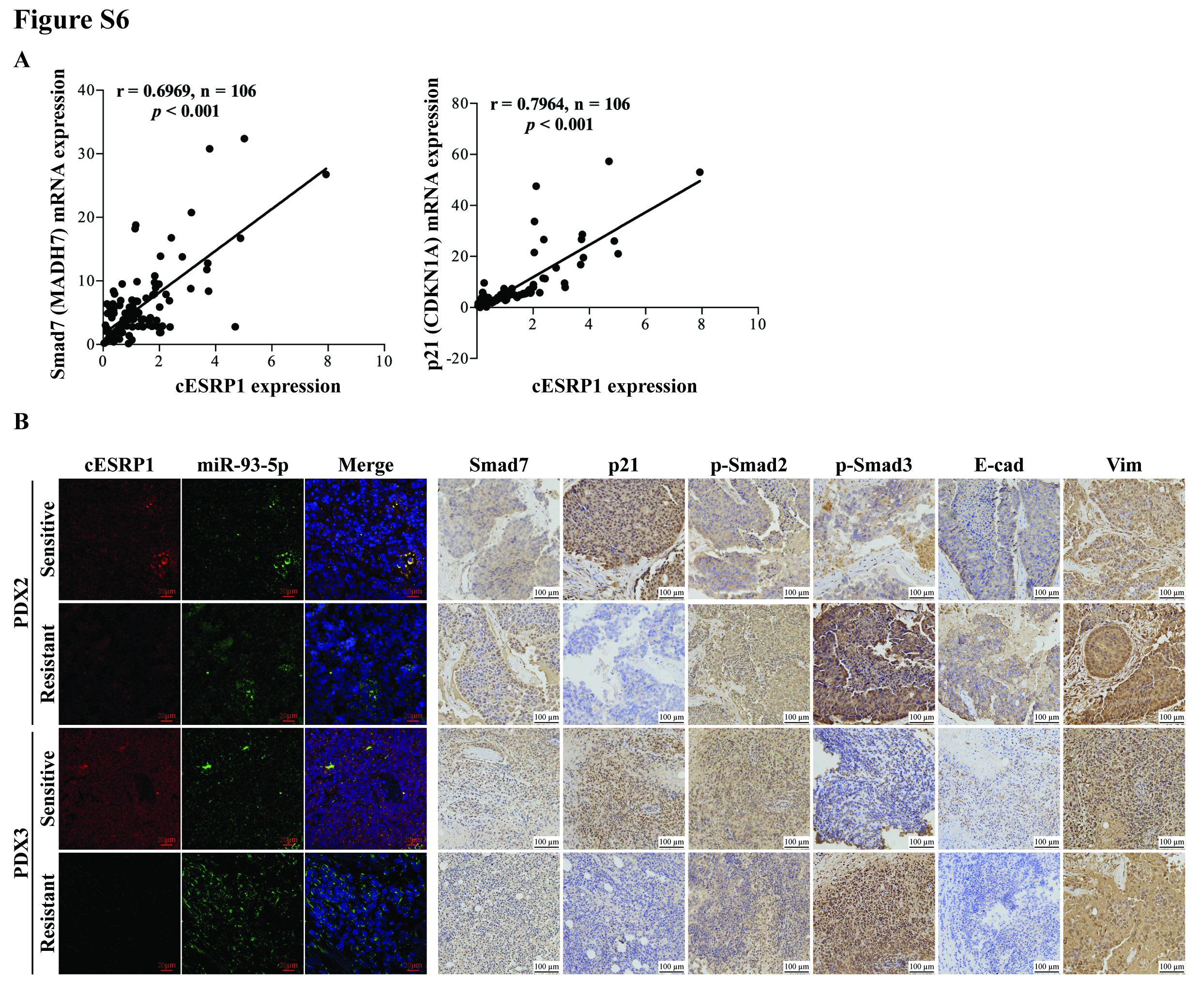

Supplement: Supplementary file 9 — Figure S6 [file 41418_2019_455_MOESM9_ESM.tif]
