# Supplementary material for: Circular RNA cESRP1 sensitises small cell lung cancer cells to chemotherapy by sponging miR-93-5p to inhibit TGF-β signalling
Source: Cell Death Differ. 2019 Nov 14;27(5):1709–27. doi: 10.1038/s41418-019-0455-x (PMC7206039; doi:10.1038/s41418-019-0455-x)
Supplement: Supplementary file 12 — Supplementary Table S3 [file 41418_2019_455_MOESM12_ESM.docx]

**Supplementary table S3. Significant differential miRNAs between H69 and H69AR cells measured by miRNA microarray (P value < 0.01)**

| **No.** | **Probe_ID** | **Sample A Signal**  **(H69)** | **Sample B Signal**  **(H69AR)** | **log2 (Sample B / Sample A)** | chromosome |
| --- | --- | --- | --- | --- | --- |
| 1 | hsa-miR-375 | 6,454.34 | 20.05 | -8.31 | 2q35 |
| 2 | hsa-miR-200b | 6,273.58 | 23.22 | -8.19 | 1p36.33 |
| 3 | hsa-miR-335* | 3,835.09 | 13.18 | -8.07 | 7q32.2 |
| 4 | hsa-miR-335 | 3,663.11 | 13.79 | -8.04 | 7q32.2 |
| 5 | hsa-miR-224 | 4.35 | 866.73 | 7.70 | Xq28 |
| 6 | hsa-miR-143 | 4.36 | 789.54 | 7.41 | 5q32 |
| 7 | hsa-miR-216b | 1,367.48 | 7.65 | -7.41 | 2p16.1 |
| 8 | hsa-miR-145 | 6.14 | 890.69 | 7.06 | 5q32 |
| 9 | hsa-miR-100 | 43.57 | 4,656.66 | 6.64 | 11q24.1 |
| 10 | hsa-miR-200c | 17,307.45 | 278.54 | -5.94 | 12p13.31 |
| 11 | hsa-miR-217 | 448.40 | 6.73 | -5.89 | 2p16.1 |
| 12 | hsa-miR-493* | 468.53 | 9.02 | -5.75 | 14q32.2 |
| 13 | hsa-miR-7 | 8,780.53 | 174.39 | -5.65 | 9q21.32 |
| 14 | hsa-miR-432 | 2,116.51 | 42.31 | -5.65 | 14q32.2 |
| 15 | hsa-miR-199a-3p | 163.81 | 7,822.89 | 5.61 | 19p13.2 |
| 16 | hsa-miR-216a | 305.13 | 7.61 | -5.33 | 2p16.1 |
| 17 | hsa-miR-487b | 593.66 | 15.51 | -5.26 | 14q32.31 |
| 18 | hsa-miR-379* | 87.59 | 2.99 | -5.22 | 14q32.31 |
| 19 | hsa-miR-99a | 31.32 | 1,161.16 | 5.21 | 21q21.1 |
| 20 | hsa-miR-199a-5p | 13.08 | 482.45 | 5.08 | 19p13.2 |
| 21 | hsa-miR-214 | 273.73 | 9,411.86 | 5.02 | 1q24.3 |
| 22 | hsa-miR-382 | 525.03 | 19.72 | -4.86 | 14q32.31 |
| 23 | hsa-miR-195 | 29.20 | 774.93 | 4.78 | 17p13.1 |
| 24 | hsa-miR-379 | 421.69 | 16.33 | -4.70 | 14q32.31 |
| 25 | hsa-miR-376a | 154.50 | 6.80 | -4.66 | 14q32.31 |
| 26 | hsa-miR-31 | 19.84 | 453.26 | 4.64 | 9p21.3 |
| 27 | hsa-miR-10a | 166.79 | 7.61 | -4.54 | 17q21.32 |
| 28 | hsa-miR-495 | 432.48 | 19.53 | -4.51 | 14q32.31 |
| 29 | hsa-miR-125b | 817.38 | 17,867.52 | 4.44 | 11q24.1 |
| 30 | hsa-miR-10b | 239.12 | 12.19 | -4.34 | 2q31.1 |
| 31 | hsa-miR-376c | 152.76 | 8.74 | -4.26 | 14q32.31 |
| 32 | hsa-miR-127-3p | 264.57 | 14.57 | -4.21 | 14q32.2 |
| 33 | hsa-miR-485-3p | 201.48 | 11.46 | -4.15 | 14q32.31 |
| 34 | hsa-miR-134 | 253.52 | 16.61 | -3.93 | 14q32.31 |
| 35 | hsa-miR-654-3p | 135.44 | 9.21 | -3.89 | 14q32.31 |
| 36 | hsa-miR-494 | 340.42 | 23.21 | -3.88 | 14q32.31 |
| 37 | hsa-miR-299-5p | 63.59 | 4.52 | -3.81 | 14q32.31 |
| 38 | hsa-miR-346 | 142.45 | 11.14 | -3.67 | 10q23.2 |
| 39 | hsa-miR-483-5p | 732.65 | 54.49 | -3.63 | 11p15.5 |
| 40 | hsa-miR-92b* | 8.74 | 83.51 | 3.35 | 1q22 |
| 41 | hsa-miR-1224-5p | 242.56 | 26.22 | -3.33 | 3q27.1 |
| 42 | hsa-miR-218 | 206.82 | 18.48 | -3.24 | 4p15.31 |
| 43 | hsa-miR-708 | 96.69 | 10.88 | -3.12 | 11q14.1 |
| 44 | hsa-miR-411* | 133.62 | 16.40 | -3.11 | 14q32.31 |
| 45 | hsa-miR-301a | 19.88 | 178.36 | 3.06 | 17q22 |
| 46 | hsa-miR-329 | 80.60 | 9.30 | -3.05 | 14q32.31 |
| 47 | hsa-miR-26b | 333.25 | 2,757.24 | 3.02 | 2q35 |
| 48 | hsa-miR-486-5p | 20.88 | 168.02 | 2.98 | 8p11.21 |
| 49 | hsa-miR-923 | 3,989.25 | 569.35 | -2.84 |  |
| 50 | hsa-miR-28-5p | 19.80 | 144.71 | 2.81 | 3q28 |
| 51 | hsa-miR-149* | 428.72 | 74.95 | -2.75 | 2q37.3 |
| 52 | hsa-miR-505* | 57.46 | 362.36 | 2.69 | Xq27.1 |
| 53 | hsa-miR-485-5p | 76.11 | 12.85 | -2.68 | 14q32.31 |
| 54 | hsa-miR-936 | 112.94 | 19.39 | -2.62 | 10q25.1 |
| 55 | hsa-miR-29a | 78.94 | 460.97 | 2.61 | 7q32.3 |
| 56 | hsa-miR-409-3p | 69.31 | 11.71 | -2.58 | 14q32.31 |
| 57 | hsa-miR-1469 | 349.41 | 58.58 | -2.57 | 15q26.2 |
| 58 | hsa-miR-24 | 879.82 | 4,759.30 | 2.45 | 9q22.32 |
| 59 | hsa-miR-765 | 92.39 | 19.76 | -2.44 | 1q23.1 |
| 60 | hsa-miR-197 | 99.74 | 495.21 | 2.39 | 1p13.3 |
| 61 | hsa-miR-99b | 489.09 | 2,103.62 | 2.11 | 19q13.41 |
| 62 | hsa-miR-125a-5p | 2,121.30 | 9,360.49 | 2.10 | 19q13.41 |
| 63 | hsa-miR-23a | 3,198.24 | 13,081.13 | 2.02 | 19p13.13 |
| 64 | hsa-miR-638 | 2,752.88 | 699.84 | -1.98 | 19p13.2 |
| 65 | hsa-miR-1275 | 2,714.17 | 677.40 | -1.97 | 6 |
| 66 | hsa-miR-27a | 515.20 | 2,034.19 | 1.96 | 19p13.13 |
| 67 | hsa-miR-21 | 7,236.65 | 26,700.40 | 1.84 | 17q23.1 |
| 68 | hsa-miR-574-5p | 297.39 | 91.84 | -1.70 | 4 |
| 69 | hsa-miR-324-5p | 40.85 | 131.84 | 1.69 | 17p13.1 |
| 70 | hsa-miR-663 | 341.14 | 128.67 | -1.53 | 20p11.1 |
| 71 | hsa-miR-222 | 583.09 | 1,544.61 | 1.39 |  |
| 72 | hsa-miR-421 | 156.91 | 367.95 | 1.32 |  |
| 73 | hsa-miR-221 | 344.44 | 801.06 | 1.26 |  |
| 74 | hsa-miR-27b | 1,254.00 | 2,937.56 | 1.21 |  |
| 75 | hsa-miR-23b | 5,303.07 | 12,176.63 | 1.20 |  |
| 76 | hsa-miR-361-5p | 2,821.27 | 1,247.87 | -1.18 |  |
| 77 | hsa-miR-877 | 352.14 | 789.34 | 1.17 |  |
| 78 | hsa-miR-183 | 1,885.45 | 4,147.32 | 1.16 |  |
| 79 | hsa-miR-1246 | 18,178.79 | 8,399.43 | -1.11 |  |
| 80 | hsa-miR-191 | 4,246.30 | 1,992.85 | -1.09 |  |
| 81 | hsa-miR-128 | 490.61 | 1,071.81 | 1.09 |  |
| 82 | hsa-miR-130b | 301.35 | 609.02 | 1.08 |  |
| 83 | hsa-miR-1180 | 124.32 | 260.40 | 1.07 |  |
| 84 | hsa-miR-15a | 321.22 | 667.43 | 1.03 |  |
| 85 | hsa-miR-92a | 27,966.93 | 14,178.41 | -1.00 |  |
| 86 | hsa-miR-132 | 270.45 | 533.41 | 0.98 |  |
| 87 | hsa-miR-20b | 4,633.50 | 2,432.38 | -0.95 |  |
| 88 | hsa-miR-92b | 16,350.76 | 8,266.02 | -0.94 |  |
| 89 | hsa-miR-374b | 1,210.23 | 617.94 | -0.93 |  |
| 90 | hsa-miR-25 | 9,060.44 | 4,780.17 | -0.92 |  |
| 91 | hsa-let-7b | 21,882.23 | 11,552.59 | -0.91 |  |
| 92 | hsa-let-7e | 25,328.08 | 13,470.29 | -0.91 |  |
| 93 | hsa-miR-1308 | 11,466.81 | 6,247.69 | -0.88 |  |
| 94 | hsa-miR-425 | 475.65 | 272.28 | -0.80 |  |
| 95 | hsa-let-7i | 8,834.50 | 15,112.88 | 0.78 |  |
| 96 | hsa-miR-106b | 1,731.98 | 2,891.30 | 0.77 |  |
| 97 | hsa-let-7c | 33,806.02 | 20,255.98 | -0.75 |  |
| 98 | hsa-let-7d | 31,363.77 | 18,654.38 | -0.75 |  |
| 99 | hsa-miR-20a | 10,936.77 | 6,714.28 | -0.68 |  |
| 100 | hsa-miR-196a | 2,828.72 | 1,772.16 | -0.66 |  |
| 101 | hsa-let-7f | 32,482.40 | 20,436.54 | -0.66 |  |
| 102 | hsa-miR-342-3p | 932.23 | 1,390.13 | 0.63 |  |
| 103 | hsa-miR-106a | 8,445.28 | 5,478.16 | -0.61 |  |
| 104 | hsa-miR-720 | 482.17 | 732.43 | 0.60 |  |
| 105 | hsa-miR-17 | 8,763.89 | 5,901.69 | -0.59 |  |
| 106 | hsa-miR-181a | 1,184.53 | 1,782.06 | 0.59 |  |
| 107 | hsa-miR-30b | 1,281.88 | 871.32 | -0.55 |  |
| 108 | hsa-miR-19b | 1,272.50 | 872.70 | -0.54 |  |
| 109 | hsa-let-7a | 38,956.19 | 26,905.20 | -0.52 |  |
| 110 | hsa-miR-9 | 30,484.05 | 21,325.89 | -0.52 |  |
| 111 | hsa-let-7g | 5,184.47 | 7,483.50 | 0.51 |  |
| 112 | hsa-miR-182 | 5,809.37 | 8,192.36 | 0.50 |  |
| 113 | hsa-miR-423-5p | 2,331.59 | 3,046.24 | 0.41 |  |
| 114 | hsa-miR-1280 | 3,304.08 | 4,351.07 | 0.39 |  |
| 115 | hsa-miR-93 | 2,208.94 | 2,880.50 | 0.39 |  |
| 116 | hsa-miR-107 | 1,519.29 | 1,981.72 | 0.39 |  |
| 117 | hsa-miR-16 | 8,048.11 | 10,336.45 | 0.34 |  |
| 118 | hsa-miR-320c | 5,008.38 | 3,991.64 | -0.32 |  |
| 119 | hsa-miR-320b | 4,280.06 | 3,403.00 | -0.29 |  |
| 120 | hsa-miR-320a | 4,926.46 | 3,890.68 | -0.29 |  |
| 121 | hsa-miR-9* | 7,778.13 | 6,630.25 | -0.25 |  |
| 122 | hsa-miR-1826 | 16,122.78 | 13,561.89 | -0.24 |  |
