# Supplementary material for: Circular RNA cESRP1 sensitises small cell lung cancer cells to chemotherapy by sponging miR-93-5p to inhibit TGF-β signalling
Source: Cell Death Differ. 2019 Nov 14;27(5):1709–27. doi: 10.1038/s41418-019-0455-x (PMC7206039; doi:10.1038/s41418-019-0455-x)
Supplement: Supplementary file 13 — Supplementary Table S4 [file 41418_2019_455_MOESM13_ESM.docx]

| **Supplementary table S4. PDX characteristics used throughout this study.** | | | | | |
| --- | --- | --- | --- | --- | --- |
| **ID** | **Source** | **Patient Diagnosis** | **PDX Diagnosis** | **Treatment** | **Site** |
| PDX1 | Zhujiang Hospital, China | SCLC | SCLC | none | Lung |
| PDX2 | Guangdong Provincial People's Hospital, China | SCLC | SCLC | C/E | lymph node |
| PDX3 | Guangdong Provincial People's Hospital, China | SCLC | SCLC | none | lymph node |
